# Supplementary material for: Transgenic female mice producing trans 10, cis 12-conjugated linoleic acid present excessive prostaglandin E2, adrenaline, corticosterone, glucagon, and FGF21
Source: Sci Rep. 2024 May 30;14:12430. doi: 10.1038/s41598-024-63282-7 (PMC11139873; doi:10.1038/s41598-024-63282-7)
Supplement: Supplementary file 1 — Supplementary Information 1. [file 41598_2024_63282_MOESM1_ESM.pdf]

Note: Before blotting, a membrane membrane was cut into 2-3 parts, the cut position was usually chosen at one of the molecular size markers nearby. Each portion was then hybridised with a corresponding antibody in a western blot test. After development of signals, the shred of membranes were restored by ImageJ software. All original, replicate blots were provided in the supplementary information file.

Fig 3f. Western blot of Brown adipose tissue

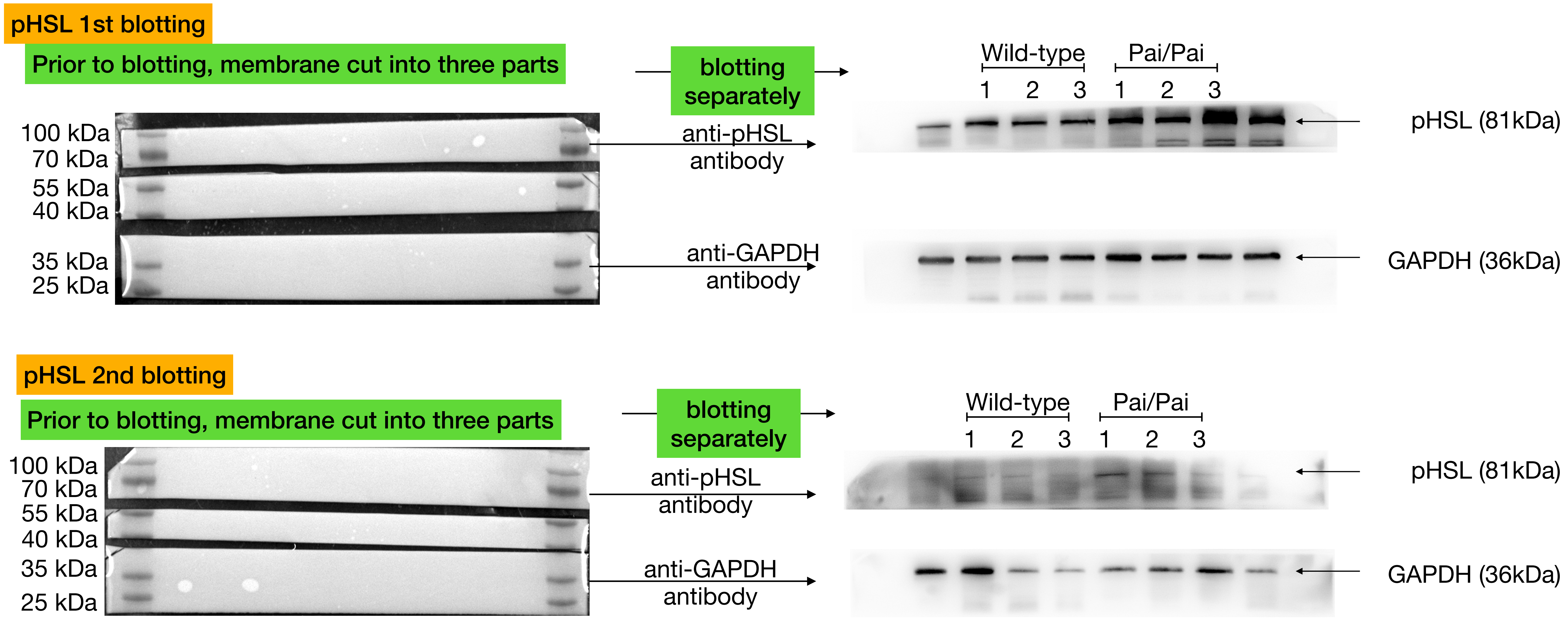

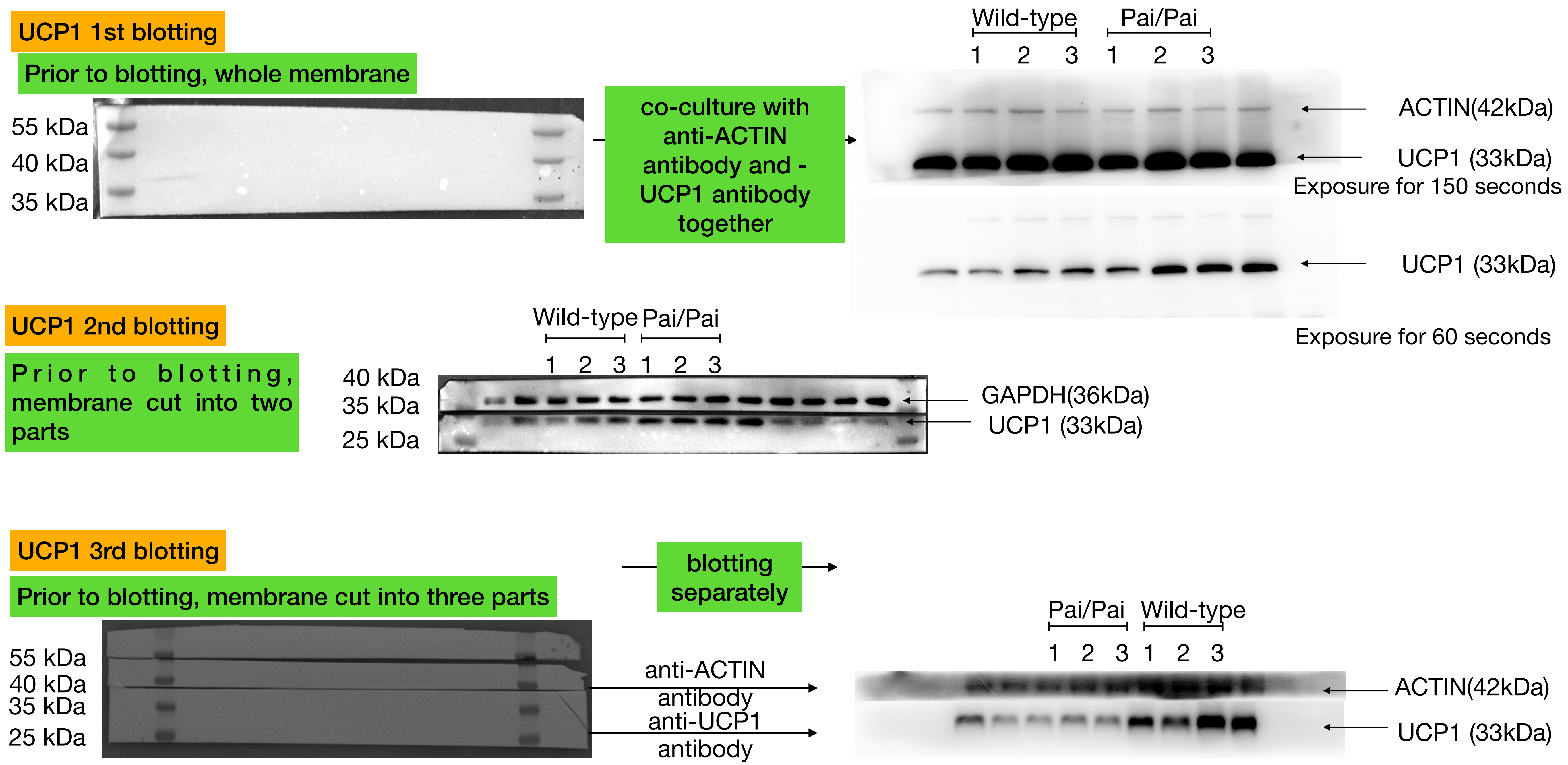
UCP1 1st blotting
Prior to blotting, whole membrane
55 kDa
40 kDa
35 kDa
co-culture with anti-ACTIN antibody and - UCP1 antibody together
Wild-type
Pai/Pai
1 2 3 1 2 3
ACTIN(42kDa)
UCP1 (33kDa)
Exposure for 150 seconds
UCP1 (33kDa)
Exposure for 60 seconds
UCP1 2nd blotting
Prior to blotting, membrane cut into two parts
Wild-type
Pai/Pai
1 2 3 1 2 3
40 kDa
35 kDa
25 kDa
GAPDH(36kDa)
UCP1 (33kDa)
UCP1 3rd blotting
Prior to blotting, membrane cut into three parts
blotting separately
55 kDa
40 kDa
35 kDa
25 kDa
anti-ACTIN antibody
anti-UCP1 antibody
Pai/Pai
Wild-type
1 2 3 1 2 3
ACTIN(42kDa)
UCP1 (33kDa)

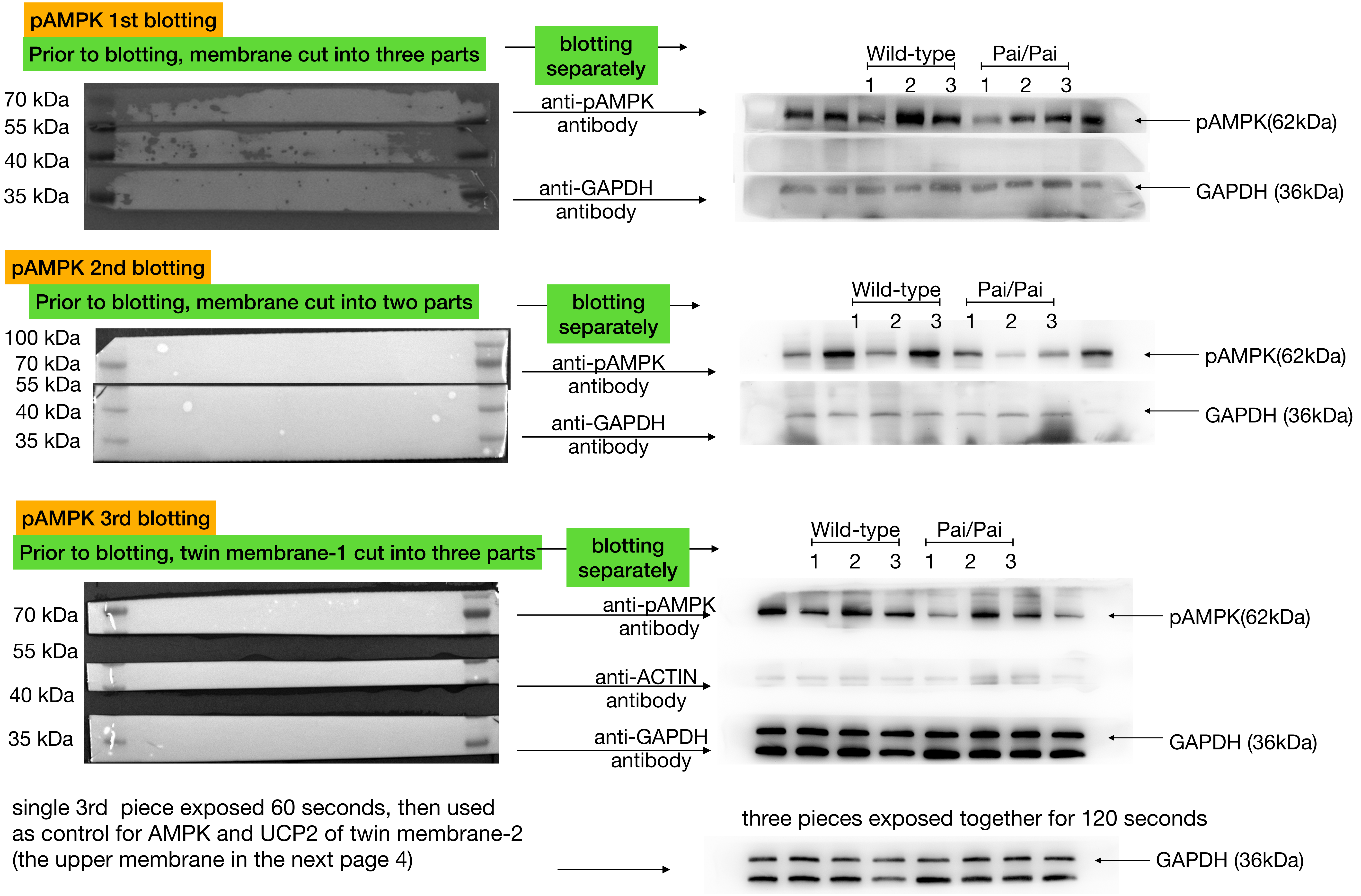
pAMPK 1st blotting
Prior to blotting, membrane cut into three parts
blotting separately
70 kDa
55 kDa
40 kDa
35 kDa
anti-pAMPK antibody
anti-GAPDH antibody
Wild-type
Pai/Pai
1 2 3 1 2 3
pAMPK(62kDa)
GAPDH (36kDa)
pAMPK 2nd blotting
Prior to blotting, membrane cut into two parts
blotting separately
100 kDa
70 kDa
55 kDa
40 kDa
35 kDa
anti-pAMPK antibody
anti-GAPDH antibody
Wild-type
Pai/Pai
1 2 3 1 2 3
pAMPK(62kDa)
GAPDH (36kDa)
pAMPK 3rd blotting
Prior to blotting, twin membrane-1 cut into three parts
blotting separately
70 kDa
55 kDa
40 kDa
35 kDa
anti-pAMPK antibody
anti-ACTIN antibody
anti-GAPDH antibody
Wild-type
Pai/Pai
1 2 3 1 2 3
pAMPK(62kDa)
GAPDH (36kDa)
single 3rd piece exposed 60 seconds, then used as control for AMPK and UCP2 of twin membrane-2 (the upper membrane in the next page 4)
three pieces exposed together for 120 seconds
GAPDH (36kDa)

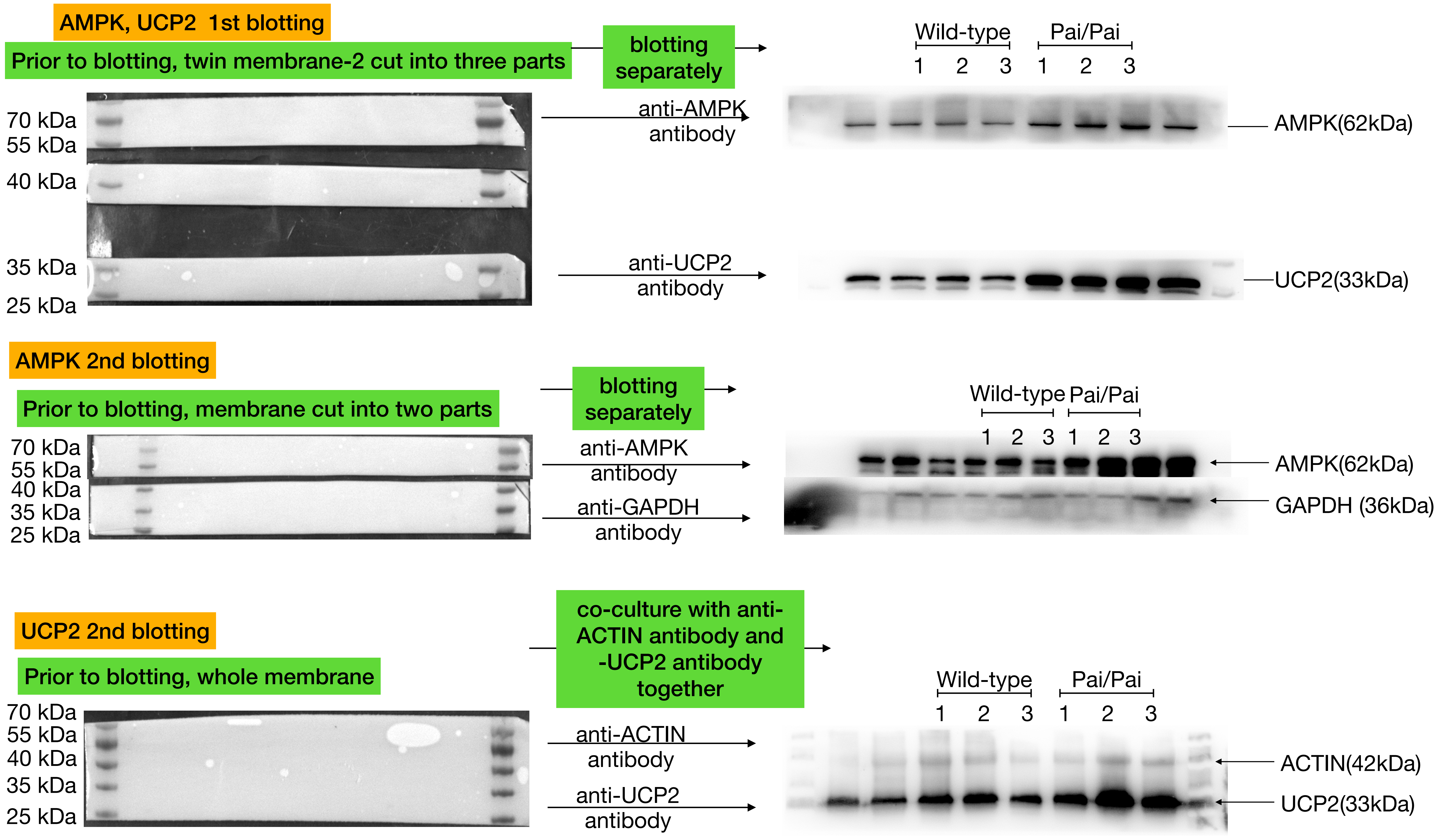

AMPK, UCP2 1st blotting
Prior to blotting, twin membrane-2 cut into three parts
blotting separately
70 kDa
55 kDa
40 kDa
35 kDa
25 kDa
anti-AMPK antibody
anti-UCP2 antibody
Wild-type
Pai/Pai
1 2 3 1 2 3
AMPK(62kDa)
UCP2(33kDa)
AMPK 2nd blotting
Prior to blotting, membrane cut into two parts
blotting separately
70 kDa
55 kDa
40 kDa
35 kDa
25 kDa
anti-AMPK antibody
anti-GAPDH antibody
Wild-type
Pai/Pai
1 2 3 1 2 3
AMPK(62kDa)
GAPDH (36kDa)
UCP2 2nd blotting
Prior to blotting, whole membrane
co-culture with anti-ACTIN antibody and -UCP2 antibody together
70 kDa
55 kDa
40 kDa
35 kDa
25 kDa
anti-ACTIN antibody
anti-UCP2 antibody
Wild-type
Pai/Pai
1 2 3 1 2 3
ACTIN(42kDa)
UCP2(33kDa)

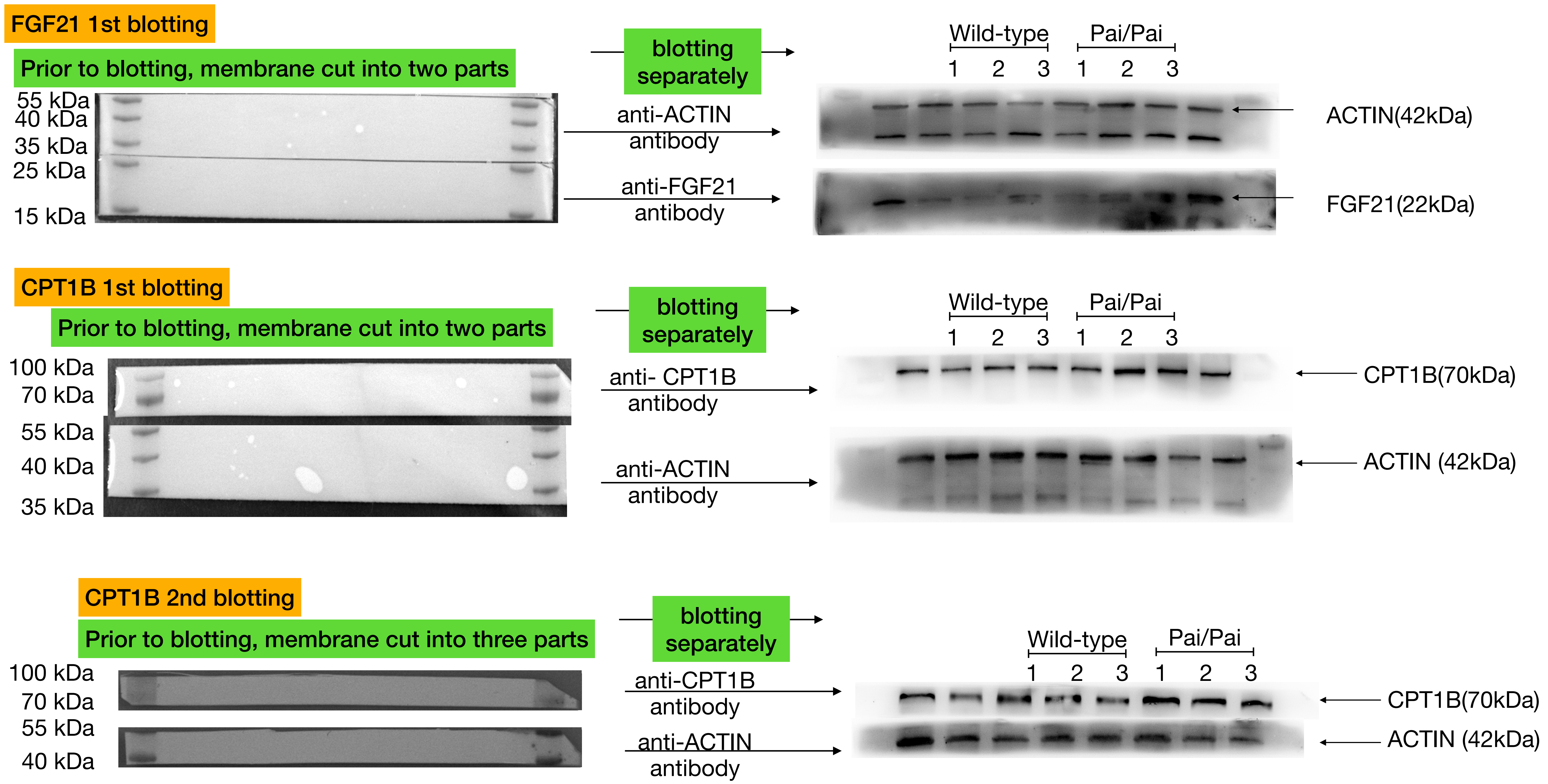
FGF21 1st blotting
Prior to blotting, membrane cut into two parts
55 kDa
40 kDa
35 kDa
25 kDa
15 kDa
blotting separately
anti-ACTIN antibody
anti-FGF21 antibody
Wild-type
Pai/Pai
1 2 3 1 2 3
ACTIN(42kDa)
FGF21(22kDa)
CPT1B 1st blotting
Prior to blotting, membrane cut into two parts
100 kDa
70 kDa
55 kDa
40 kDa
35 kDa
blotting separately
anti- CPT1B antibody
anti-ACTIN antibody
Wild-type
Pai/Pai
1 2 3 1 2 3
CPT1B(70kDa)
ACTIN (42kDa)
CPT1B 2nd blotting
Prior to blotting, membrane cut into three parts
100 kDa
70 kDa
55 kDa
40 kDa
blotting separately
anti-CPT1B antibody
anti-ACTIN antibody
Wild-type
Pai/Pai
1 2 3 1 2 3
CPT1B(70kDa)
ACTIN (42kDa)

Fig 5b. Western blot of livers

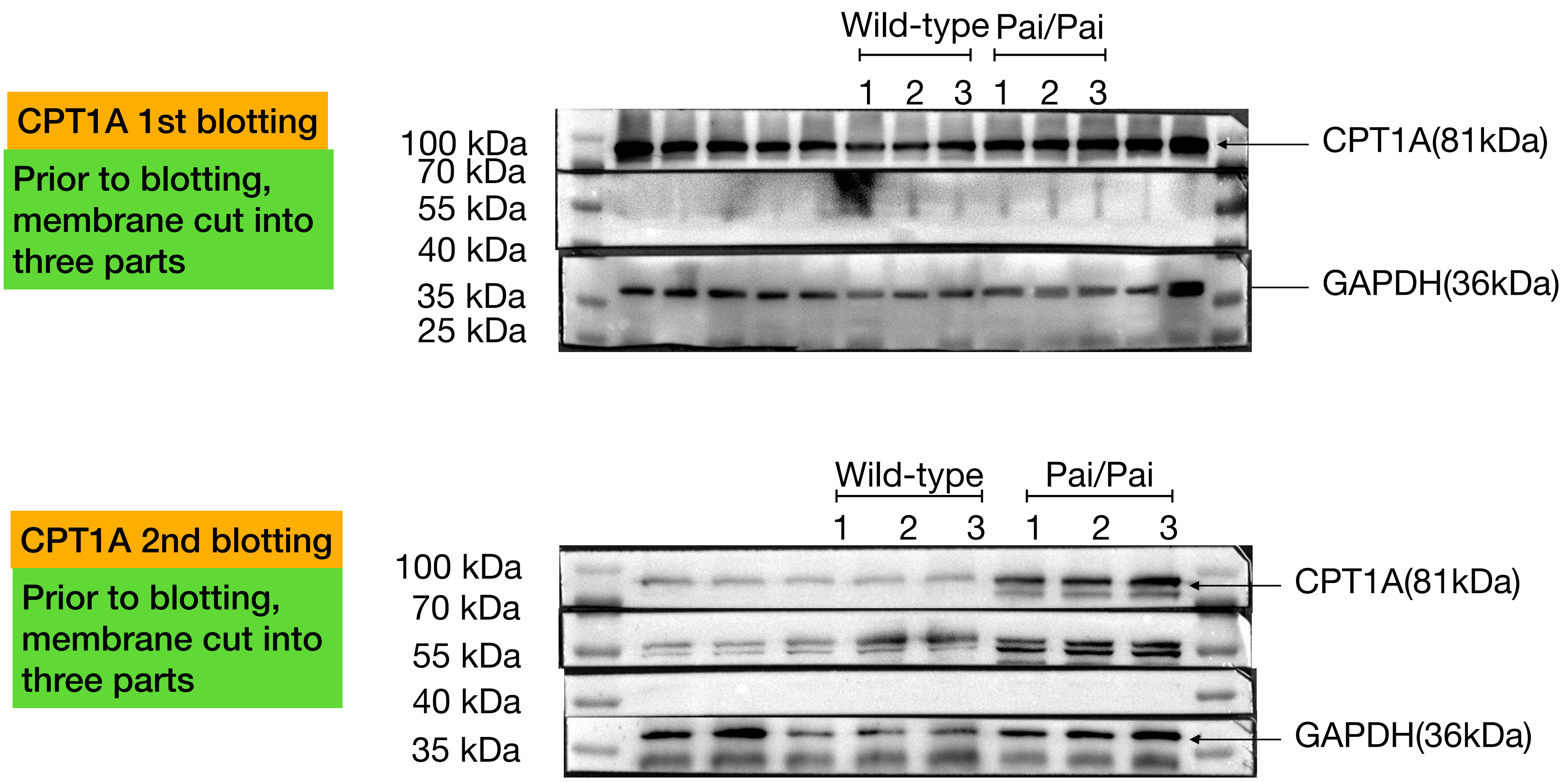

AMPK 1st blotting

Prior to blotting, membrane cut into two parts

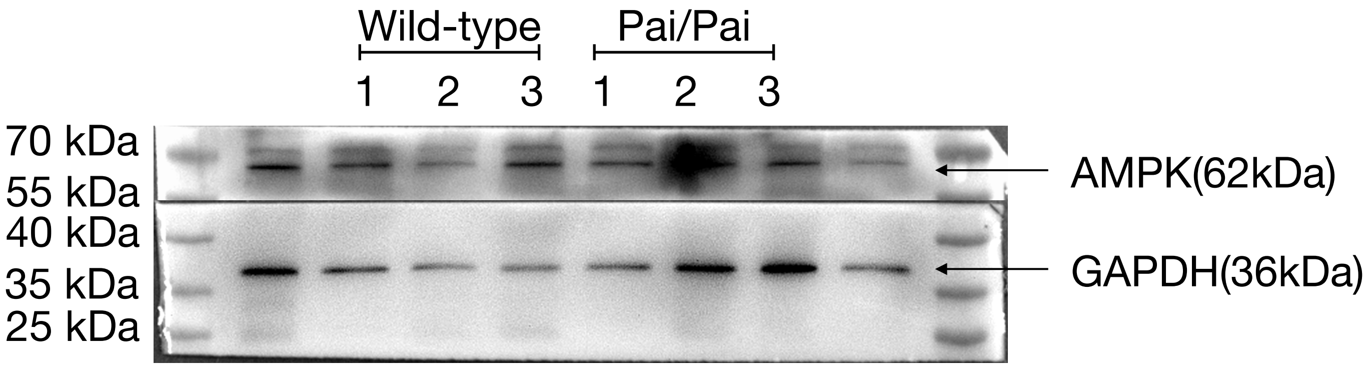

AMPK 2nd blotting

Prior to blotting, membrane cut into four parts

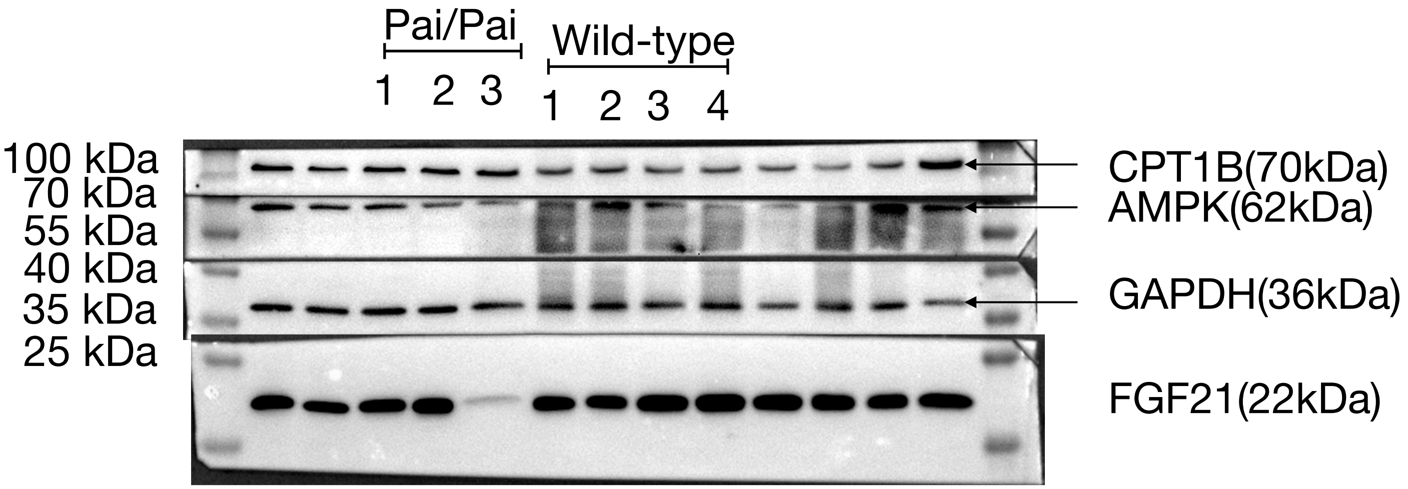

pAMPK 1st blotting

Prior to blotting, membrane cut into two parts

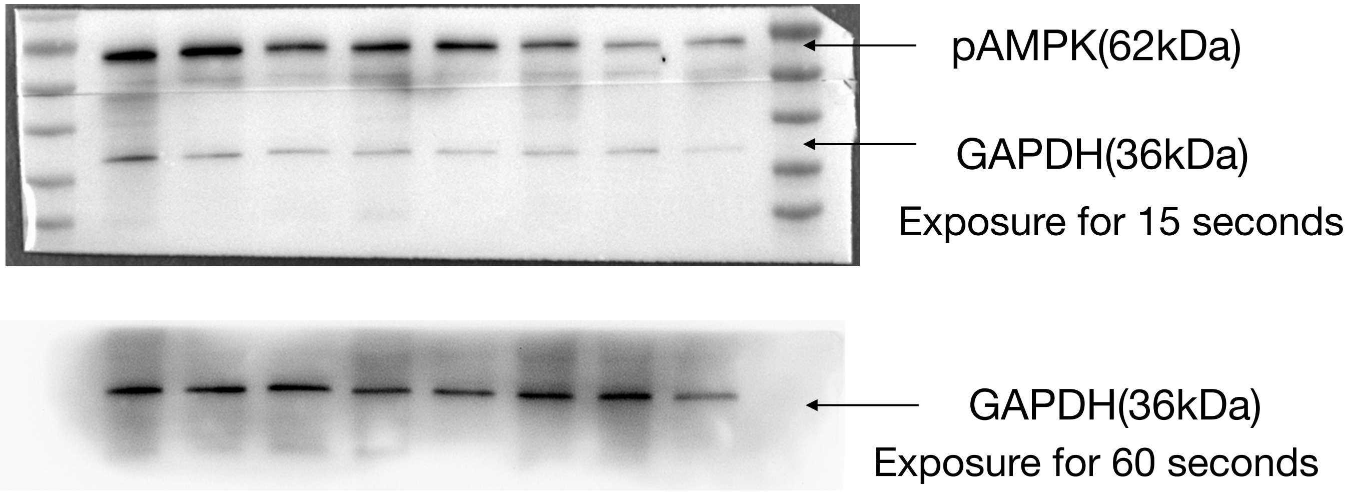

pAMPK 2nd blotting

Prior to blotting, membrane cut into three parts

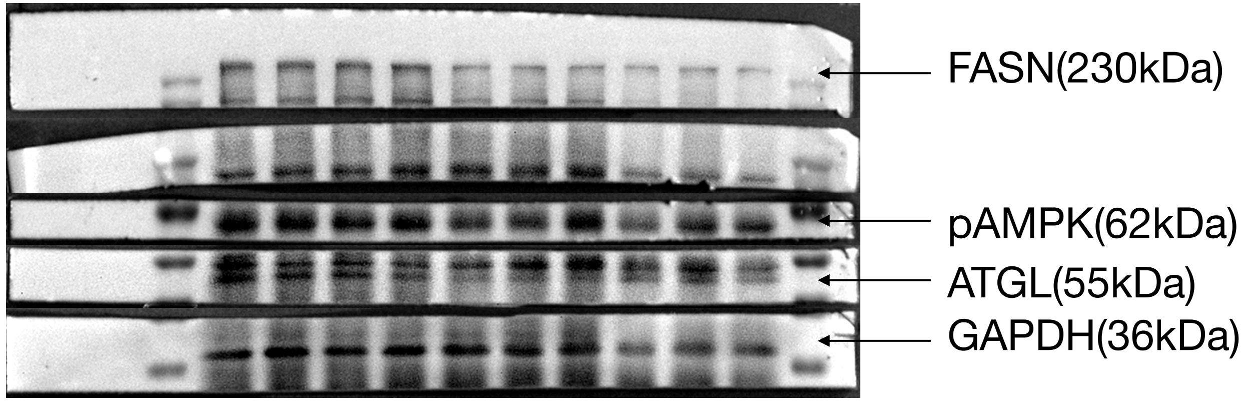

CPT1B 1st blotting

Prior to blotting, membrane cut into three parts

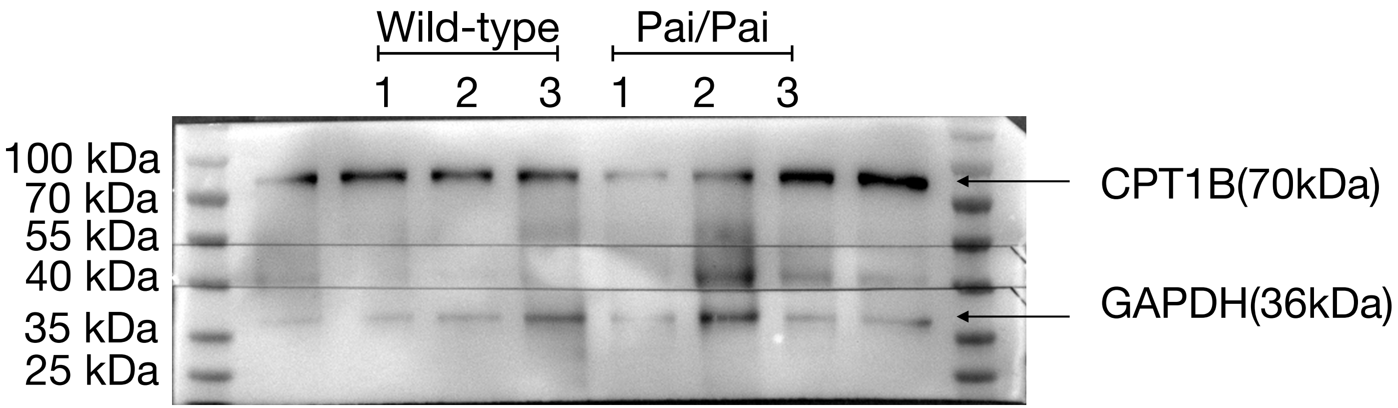

CPT1B 2nd blotting

Prior to blotting, membrane cut into three parts

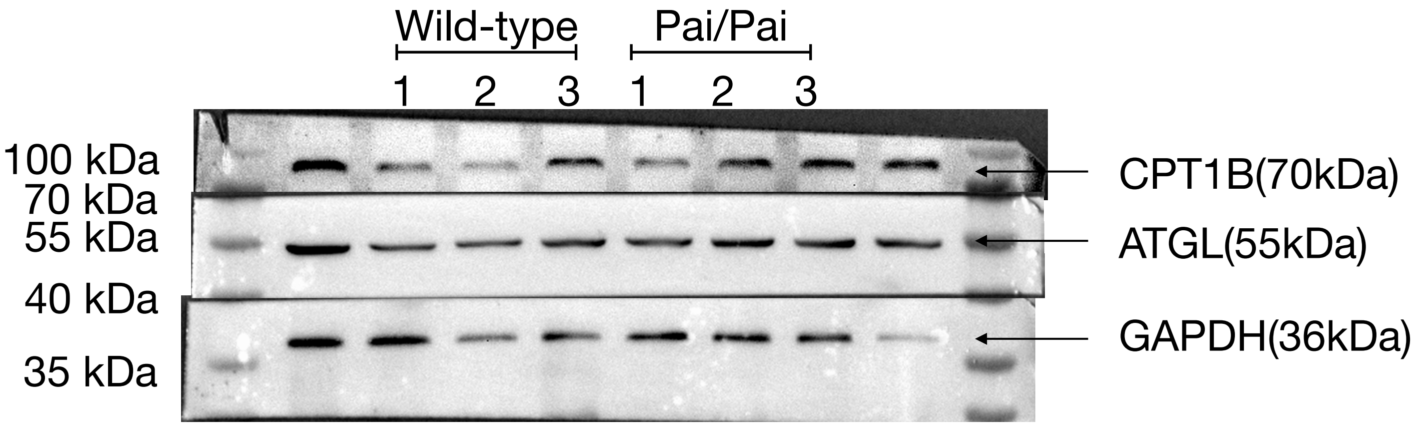

CPT1B 3rd blotting

Prior to blotting, membrane cut into four parts

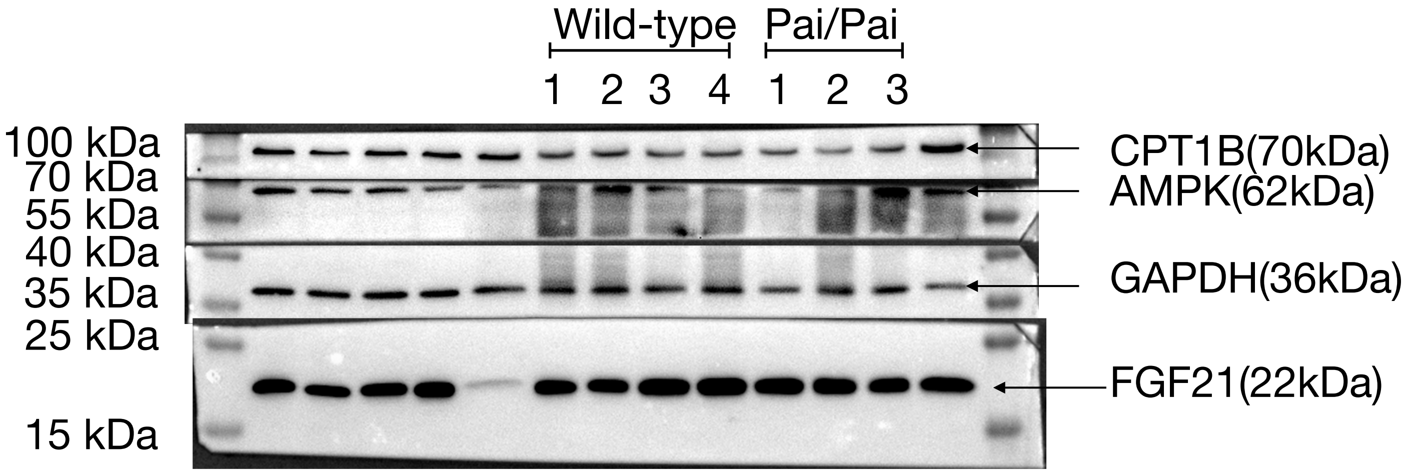

ATGL 1st blotting

Prior to blotting, membrane cut into three parts

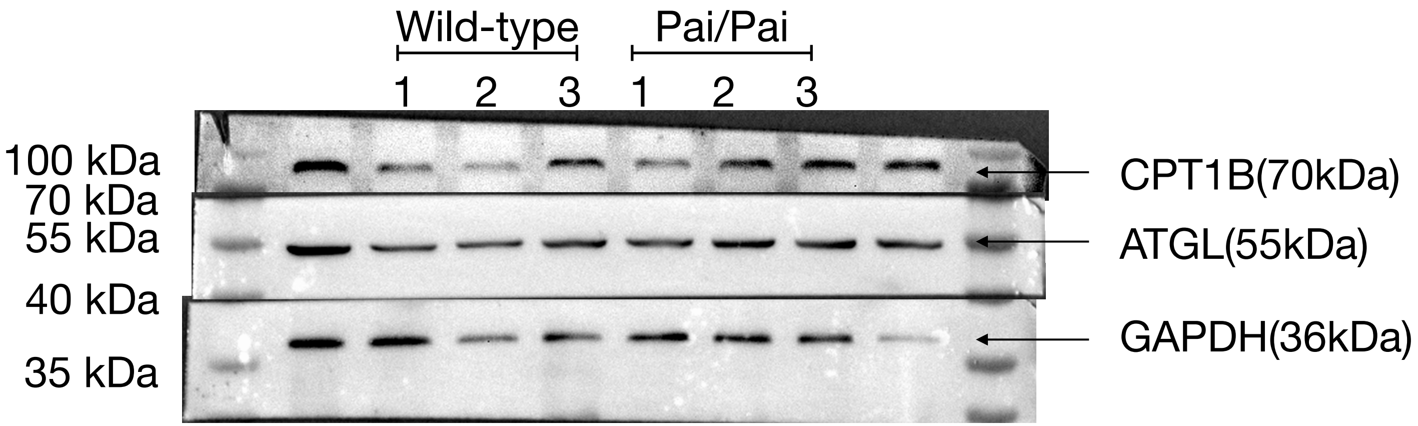

ATGL 2nd blotting

Prior to blotting, membrane cut into three parts

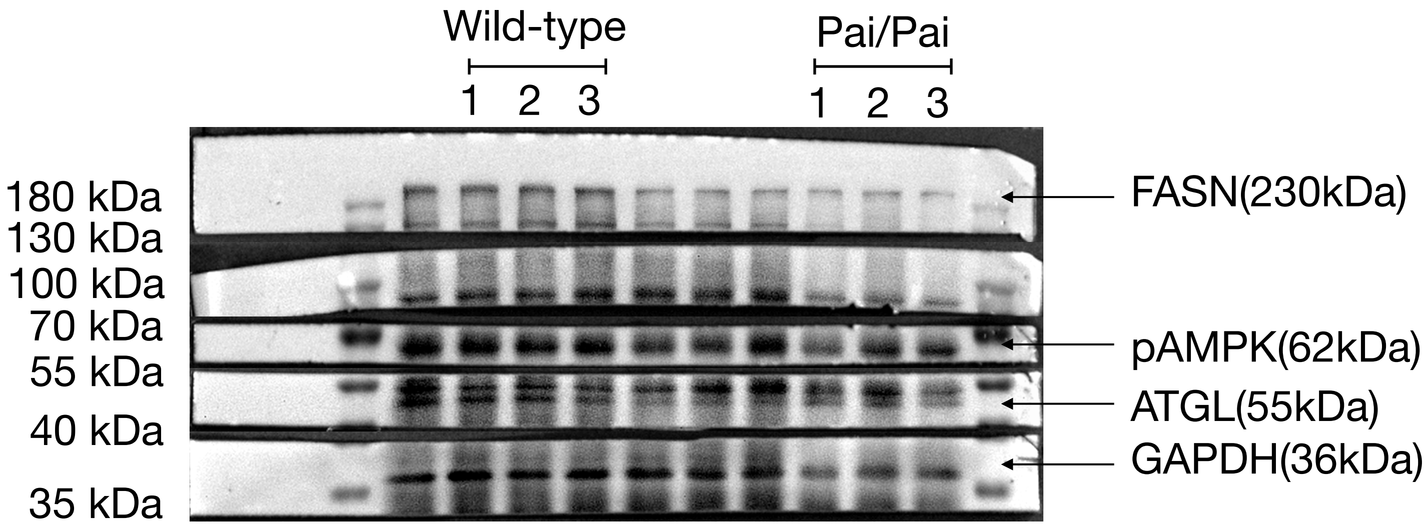

FGF21 1st blotting

Prior to blotting, membrane cut into four parts

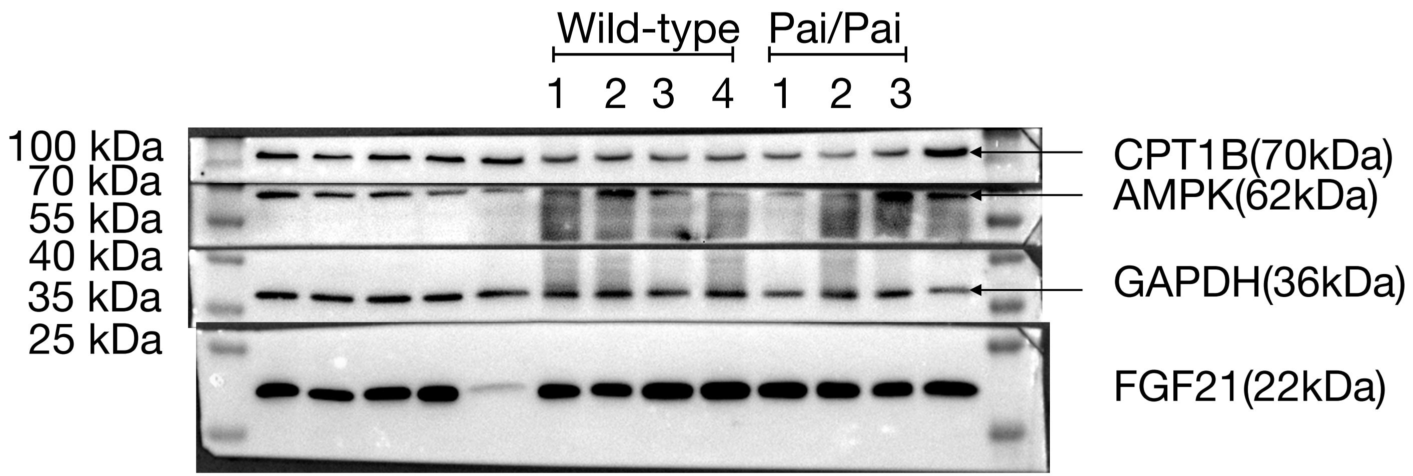

FASN 1st blotting

Prior to blotting, membrane cut into three parts

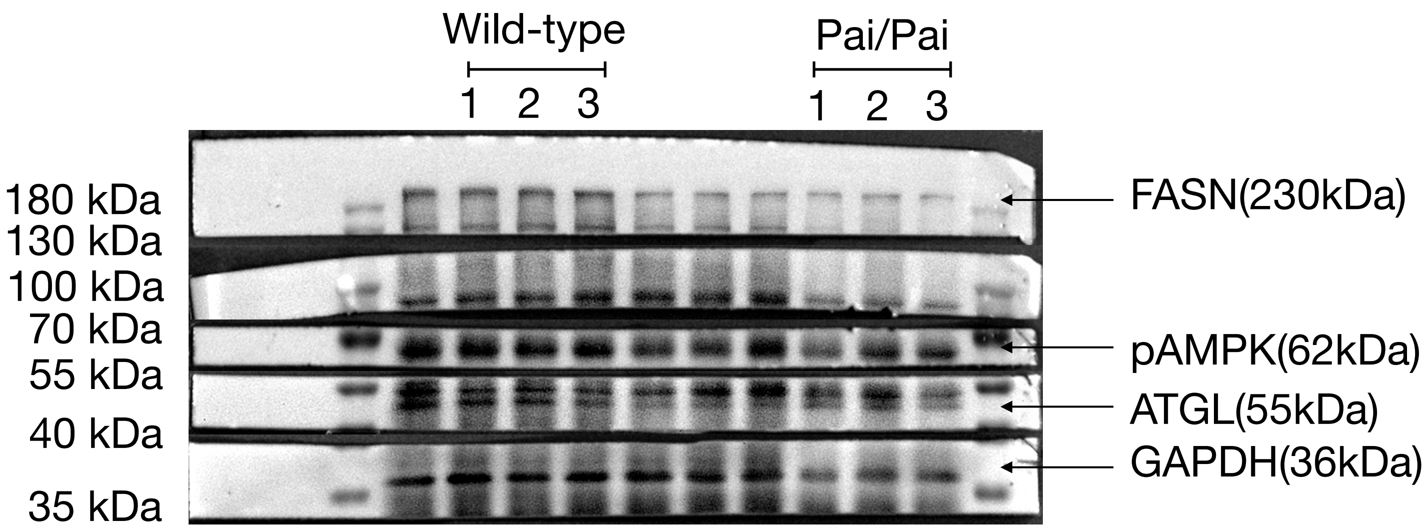

FASN 2nd blotting

Prior to blotting, membrane cut into three parts

Pai/Pai Wild-type

1 2 3 1 2 3

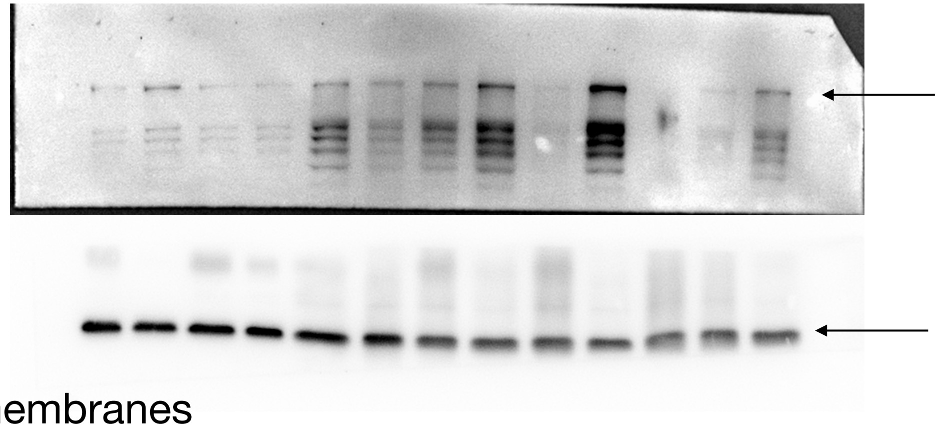

FASN(230kDa)

GAPDH(36kDa)

marker from its twin membranes

Pai/Pai Wild-type

1 2 3 1 2 3

100 kDa
70 kDa
55 kDa
40 kDa
35 kDa

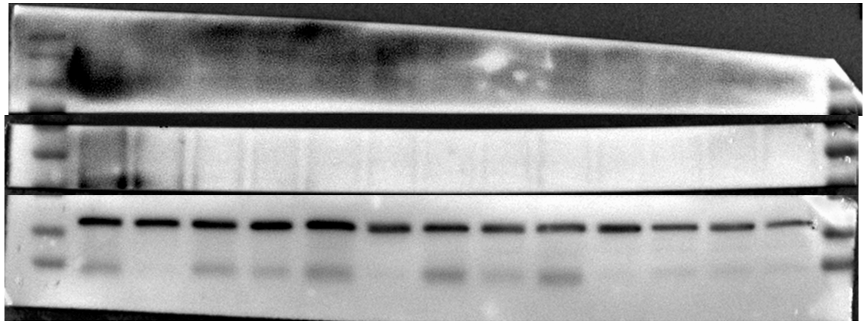

GAPDH(36kDa)

Supplementary Figure S2b. Western blot of hypothalamus

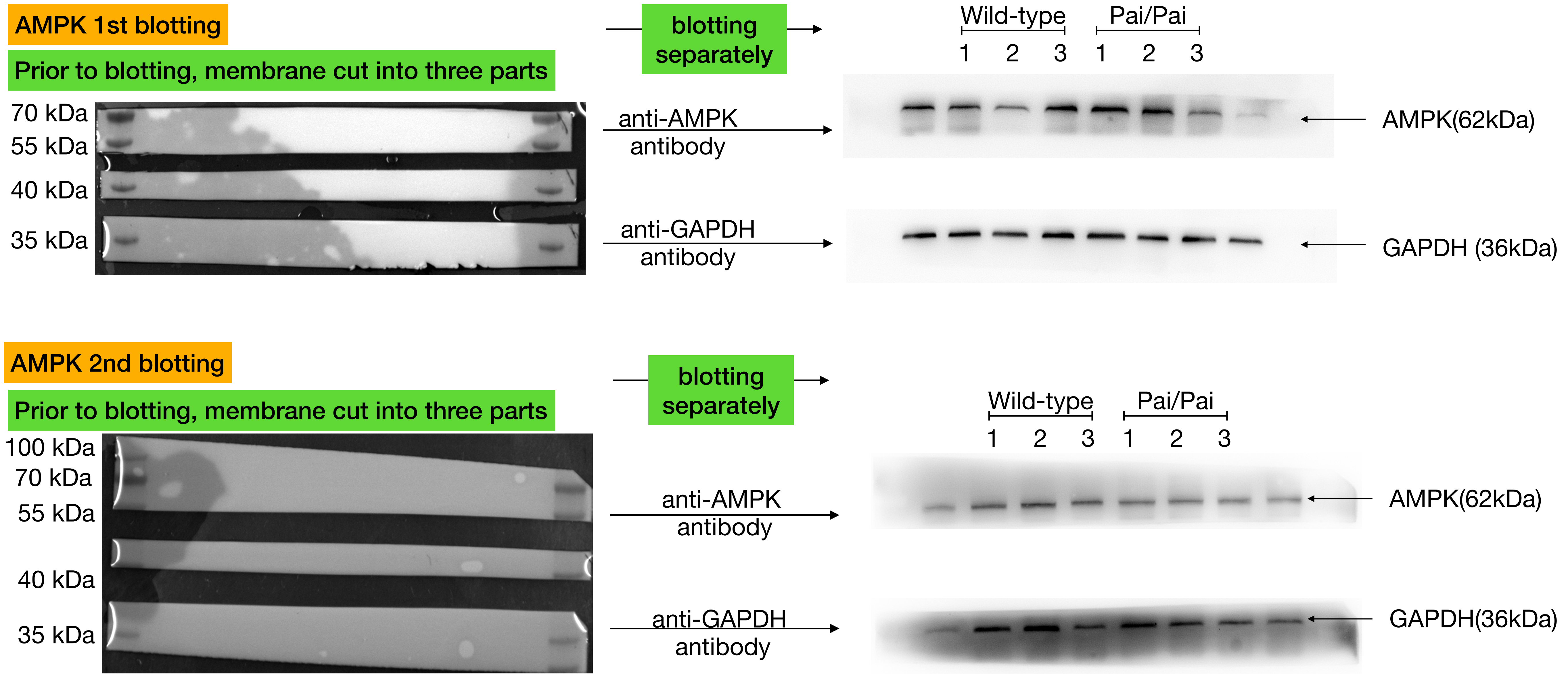

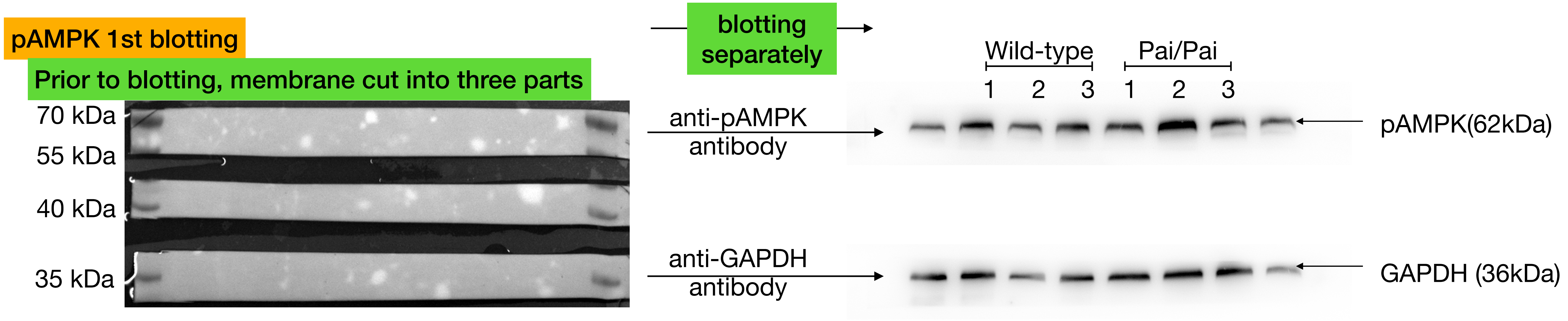
pAMPK 1st blotting
Prior to blotting, membrane cut into three parts
70 kDa
55 kDa
40 kDa
35 kDa
blotting separately
anti-pAMPK antibody
anti-GAPDH antibody
Wild-type
Pai/Pai
1 2 3 1 2 3
pAMPK(62kDa)
GAPDH (36kDa)

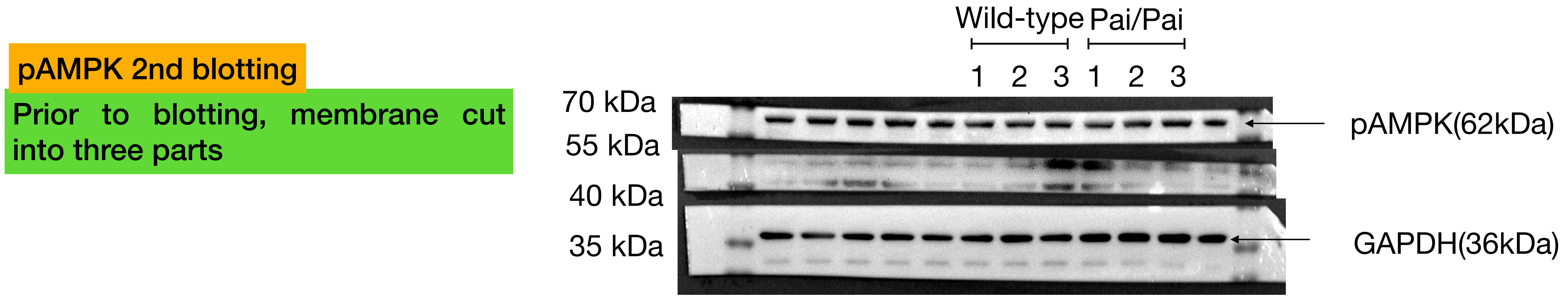
pAMPK 2nd blotting
Prior to blotting, membrane cut into three parts
Wild-type
Pai/Pai
1 2 3 1 2 3
70 kDa
55 kDa
40 kDa
35 kDa
pAMPK(62kDa)
GAPDH(36kDa)

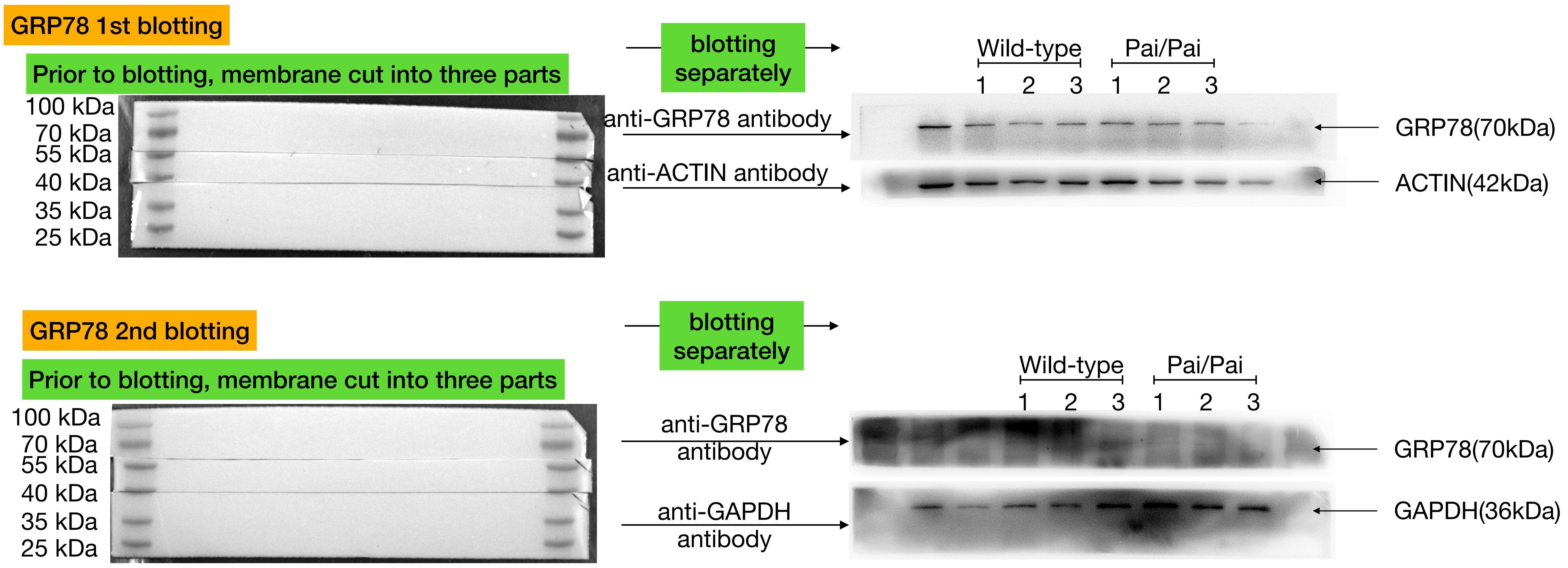
GRP78 1st blotting
Prior to blotting, membrane cut into three parts
100 kDa
70 kDa
55 kDa
40 kDa
35 kDa
25 kDa
blotting separately
anti-GRP78 antibody
anti-ACTIN antibody
Wild-type
Pai/Pai
1 2 3 1 2 3
GRP78(70kDa)
ACTIN(42kDa)
GRP78 2nd blotting
Prior to blotting, membrane cut into three parts
100 kDa
70 kDa
55 kDa
40 kDa
35 kDa
25 kDa
blotting separately
anti-GRP78 antibody
anti-GAPDH antibody
Wild-type
Pai/Pai
1 2 3 1 2 3
GRP78(70kDa)
GAPDH(36kDa)
