# Supplementary material for: Transgenic female mice producing trans 10, cis 12-conjugated linoleic acid present excessive prostaglandin E2, adrenaline, corticosterone, glucagon, and FGF21
Source: Sci Rep. 2024 May 30;14:12430. doi: 10.1038/s41598-024-63282-7 (PMC11139873; doi:10.1038/s41598-024-63282-7)
Supplement: Supplementary file 2 — Supplementary Information 2. [file 41598_2024_63282_MOESM2_ESM.pdf]

Transgenic female mice producing *trans* 10, *cis* 12-conjugated linoleic acid present excessive prostaglandin E2, adrenaline, corticosterone, glucagon, and FGF21

Yu Rao[1,2,#], Lu-Wen Liang[1,#], Mei-Juan Li[3,#], Yang-Yang Wang[1], Bao-Zhu Wang[1], Ke-Mian Gou[1,2,*]

[1]*Institute of Comparative Medicine,* [2]*Jiangsu Co-innovation Center for Prevention and Control of Important Animal Infectious Diseases and Zoonoses, Department of Experimental Zoology, College of Veterinary Medicine, Yangzhou University, Yangzhou 225009, China;*

[3]*Institute of Animal Husbandry and Veterinary Science, Guizhou Academy of Agricultural Sciences, Guiyang 550005, China.*

# These authors contributed equally to this work.

***Corresponding author:** Kemian Gou, College of Veterinary Medicine, Yangzhou University, Wenhui South Road, Yangzhou 225009, China.

Email: gou@yzu.edu.cn

Supplementary data

Supplementary Tables

Supplementary Table S1. Sources of dietary ingredients

Ingredients	Grams	Sources
Lactic casein	200.0	Gansu Hualing Dairy Co. Ltd, Hezuo city, China
L-cystine	3.0	Zhongtuan shihua Food Reagents Co. Ltd, Hangzhou, China
Sucrose	176.8	Jinhuitaiya, Chemcal Reagents Co. Ltd, Tianjin, China
Corn Starch	452.2	Zhucheng Xingmao Corn Co. Ltd, Weifang city, China
Maltodextrin 10	75.0	Dongzhu Food Co. Ltd, Guilin city, China
Cellulose	50.0	Yisheng Kangyuan Biotech Co. Ltd, Zibo city, China
Soybean oil	25.0	Shandong Luhua Co. Ltd, Leiyang, China
Lard	20.0	Yaodong Baofengyuan Co. Ltd, Qingdao, China
Mineral Mix (20x)	50.0	Mingxin Chemical Reagents Co. Ltd, Zhenzhou, China
Choline bitartrate	2.0	Zhejiang Linuo Biotech Co. Ltd, Yiwu, China
Vitamin Mix (1000x)	1.0	Mingxin Chemical Reagents Co. Ltd, Zhenzhou, China
Total Gram	1055	

Supplementary Table S2. Primer sequences of real-time PCR

mRNA	Full name	Forward (top) and reverse (bottom) primers (5′ to 3′)	Genbank accession no.
36B4	Ribosomal protein, large, P0	CACTGGTCTAGGACCCGAGAAG; GGTGCCTCTGGAGATTTTCG	NM_007475.5
Acaa2	Acetyl-CoA acyltransferase 2	CTGCTACGAGGTGTGTTCATC; AGCTCTGCATGACATTGCCC	NM_177470.3
Acbp	Acyl-CoA-binding protein	CATCCGTATCACCTCACC; GTATTTACATCGCCCACA	NM_007830.4
Acox1	Acyl-CoA oxidase 1, transcript variant 1	AGATTGGTAGAAATTGCTGCAAAA; ACGCCACTTCCTTGCTCTTC	NM_015729.3
Acox2	Acyl-CoA oxidase 2, transcript variant 1	AACCCAGGGGATCGAGTGT; CGCAGCTCAGTGTTTGGGAT	NM_053115.2
Adcy3	Adenylate cyclase 3, transcript variant 1	AGATGTTCGGTGCCACCTG; ACTTCACCAGGGCTTCGTAAG	NM_138305.3
	Adiponectin	AGCCGCTTATATGTATCGCTCA; TGCCGTCATAATGATTCTGTTGG	NM_009605.5
Agpat1	1-acylglycerol-3-phosphate O-acyltransferase 1	GCTGGCTGGCAGGAATCAT; GTCTGAGCCACCTCGGACAT	NM_018862
Agpat2	1-acylglycerol-3-phosphate O-acyltransferase 2	TTTGAGGTCAGCGGACAGAA; AGGATGCTCTGGTGATTAGAGATGA	NM_026212
Agrp	Agouti related neuropeptide	GGCCTCAAGAAGACAACTGC; GACTCGTGCAGCCTTACACA	NM_007427.3
Ampk	AMP-activated protein kinase	TTGACGATGAGGCTGTGAAG; ATAAGCCACTGCAAGCTGGT	NM_178143.2
ApoB	Apolipoprotein B	CGTGGGCTCCAGCATTCTA; TCACCAGTCATTTCTGCCTTTG	NM_009693.2
Atgl	Adipose triglyceride lipase	TTCACCATCCGCTTGTTGGAG; AGATGGTCACCCAATTTCCTC	NM_025802.3
Avp	Arginine vasopressin	GCTCAACACTACGCTCTC; CTTGGGCAGTTCTGGAAG	NM_009732.2
Cart	Cocaine- and amphetamine-regulated transcript	GTCCCACGAGAAGGAGCTGCCAA; GCCCATCCGCTCTCTGAGGGG	NM_013732.7
Cd11c	CD11 antigen-like family member C, integrin alpha X	CTGGATAGCCTTTCTTCTGCTG; GCACACTGTGTCCGAACTC	NM_021334.3
Cd36	CD36 antigen, transcript variant 1	GGACATTGAGATTCTTTTCCTCTG; GCAAAGGCATTGGCTGGAAGAAC	NM_001159558.1
Cd68	CD68 antigen	CTTCCCACAGGCAGCACAG; AATGATGAGAGGCAGCAAGAGG	NM_001291058.1

Cebpβ	CCAAT/enhancer binding protein (C/EBP), beta	CTGCGGGGTTGTTGATGT; ATGCTCGAAACGGAAAAGGT	NM_001287738.1
Cgi-58	Comparative gene identification 58	TGGATTCTTGGCTGCTGCTTAC; TTAAAGGGAGTCAATGCTGCTC	NM_026179.2
Chrebp	Carbohydrate response element binding protein	CCTTCGCCAACTCAGCACTT; TGGCTTGCTCAGGCACAA	NM_021455
Cpt1a	Carnitine palmitoyltransferase 1a	CACCAACGGGCTCATCTTCTA; CAAAATGACCTAGCCTTCTATCGAA	NM_013495
Crh	Corticotropin releasing hormone	GGCATCCTGAGAGAAGTCCC; GTTAGGGGCGCTCTCTTCTC	NM_205769.3
Cypb	Cyclophilin B	TGGAGAGCACCAAGACAGACA; TGCCGGAGTCGACAATGAT	NM_011149.2
Deptor	DEP domain containing MTOR-interacting protein	ATAGACGGCACCATCTCAAAAC; GTCGGCTAATTTCTGCATGAGT	NM_145470.3
Dgat1	Diacylglycerol acyltransferase 1	GAGGCCTCTCTGCCCCTATG; GCCCCTGGACAACACAGACT	NM_010046
Dgat2	Diacylglycerol acyltransferase 2	CCGCAAAGGCTTTGTGAAG; GGAATAAGTGGGAACCAGATCA	NM_026384
Dicer	Ribonuclease type III	CACACGCCTCCTACCACTACAACAC; CCGTGGGTCTTCATAAAGGT	NM_148948.2
Dnmt1	DNA (cytosine-5)-methyltransferase 1	GTCGGACAGTGACACCCTTT; TGGGTTTCCGTTTAGTGGGG	NM_001199431.1
F4/80	Adhesion G protein-coupled receptor E1	CTTTGGCTAAGGGCTTCCAGTC; GCAAGGAGGACAGAGTTTATCGTG	NM_010130.4
Fads1	Fatty acid desaturase 1	AGCACATGCCATACAACCATC; TTTCCGCTGAACCACAAAATAGA	NM_146094.2
Fads2	Fatty acid desaturase 2	GATGGCTGCAACATGACTATGG; GCTGAGGCACCCTTTAAGTGG	NM_019699.2
Fasn	Fatty acid synthase	GCTGCGGAAACTTCAGGAAAT; AGAGACGTGTCACTCCTGGACTT	NM_007988.3
Fgf21	Fibroblast growth factor 21	CTGCTGGGGGTCTACCAAG; CTGCGCCTACCACTGTTCC	NM_020013.4
Foxa2	Forkhead box protein a2	ACTTTGGGAGAGCTTTGAGGAA; CCCATCTATTTAGGACACAGACA	NM_010446.3
Foxc2	Forkhead box protein C2	AAAGCGCCCCTCTCTCAGA;CTCAAAC TGAGCTGCGGATAAGT	NM_013519.2
G6p	Glucose-6-phosphatase	TGGGCAAAATGGCAAGGA; TCTGCCCCAGGAATCAAAAAT	NM_008061.4
G6pd	Glucose-6-phosphate dehydrogenase	GAACGCAAAGCTGAAGTGAGACT; TCATTACGCTTGCACTGTTGGT	NM_019468.2
Gapdh	Glyceraldehyde-3-phosphate de-	GAACATCATCCCTGCATCC; CCAGTGAGCTTCCCGTTCA	NM_001289726.1

	hydrogenase, transcript variant 1		
Gck	Glucokinase	CCGTGATCCGGGAAGAGAA; GGGAAACCTGACAGGGATGAG	NM_010292.5
Ghsr	ghrelin receptor	GGACCAGAACCACAAACAGACA; CAGCAGAGGATGAAAGCAAACA	NM_177330.4
Glut4	Glucose transporter type 4, transcript variant 1	GTGACTGGAACACTGGTCCTA; CCAGCCACGTTGCATTGTAG	NM_009204.2
Gnrh	Gonadotropin releasing hormone 1, transcript variant 1	CACTGGTCCTATGGGTTGC; TTCTGCCTGGCTTCCTCT	NM_008145.3
Gpat1	Glycerol-3-phosphate acyltrans-ferase 1, transcript variant 1	CAACACCATCCCCGACATC; GTGACCTTCGATTATGCGATCA	NM_001356285.1
Grp78	Glucose-regulated protein 78, transcript variant 1	ACTTGGGGACCACCTATTCCT; ATCGCCAATCAGACGCTCC	NM_001163434.1
H19	H19, transcript variant 1	GGAATGTTGAAGGACTGAGGG; GTAACCGGGATGAATGTCTGG	NR_130973.1
Hmgcr	HMG-CoA reductase, transcript variant 1	CTTGTGGAATGCCTTGTGATTG; AGCCGAAGCAGCACATGAT	NM_008255.2
Hsl	Hormone-sensitive lipase, transcript variant 1	GGAGCACTACAAACGCAACGA; TCGGCCACCGGTAAAGAG	NM_010719.5
Htgl	Hepatic triglyceride lipase, transcript variant 1	ATGGGAAATCCCCTCCAAATCT; GTGCTGAGGTCTGAGACGA	NM_008280.2
Igf1	Insulin-like growth factor 1, transcript variant 1	GCCCCACTGAAGCCTACAAA; TGAGTCTTGGGCATGTCAGTGT	NM_010512.5
Igf2r	Insulin-like growth factor 2 receptor	GCCTCTGGAGACATGAGGAC; GGTGCCTACTTTCCTGCTGA	NM_010515.2
Igfbp1	Insulin-like growth factor binding protein 1	ATCAGCCCATCCTGTGGAAC; TGCAGCTAATCTCTCTAGCACTT	NM_008341.4
Insig1	Insulin induced gene 1	TCACAGTGACTGAGCTTCAGCA; TCATCTTCATCACACCCAGGAC	NM_153526.5
Insig2a	Insulin induced gene 2a, transcript variant 2	CCCTCAATGAATGTACTGAAGGATT; TGTGAAGTGAAGCAGACCAATGT	NM_178082.3
Insr	Insulin receptor, transcript variant 1	CGAGTGCCCGTCTGGCTATA; GGCAGGGTCCCAGACATG	NM_010568.3
Irs1	Insulin receptor substrate-1	TCACAGCAGAATGAAGACC; CTACTGATGAGGAAGATATGAGG	NM_010570.4
Irs2	Insulin receptor substrate-2	GGAGAACCCAGACCCTAAGCTACT; GATGCCTTTGAGGCCTTCAC	NM_001081212.2
Lcad	Long-chain acyl-CoA dehydrogenase	TCAATGGAAGCAAGGTGTTCA; GCCACGACGATCACGAGAT	NM_007381.4

Lchad	Long-chain 3-hydroxyacyl-CoA dehydrogenase	TGCATTTGCCGCAGCTTTAC; GTTGGCCCAGATTTCGTTCA	NM_178878.3
Ldlr	Low density lipoprotein receptor, transcript variant 1	AGGCTGTGGGCTCCATAGG; TGCGGTCCAGGGTCATCT	NM_010700.3
Lepr	Leptin receptor, transcript variant 1	TGGTCCCAGCAGCTATGGT; ACCCAGAGAAGTTAGCACTGT	NM_146146.3
Lkb1	Liver kinase B1, transcript variant 2	GACTTCACAGTGCCTGGTGTC; AGCAGACAGGGAGCTACACTA	NM_001301853.2
Lpl	Lipoprotein lipase	ACTATGTGTCTAACTGCCACTTCAA; ATACATTCCCGTTACCGTCCAT	NM_008509.2
Lxrα	Liver X receptor α, transcript variant 1	TACGTCTCCATCAACCACCCC; ACTTGCTCTGAATGGACGCTG	NM_013839.4
Lxrβ	Liver X receptor β, transcript variant 1	GCAGTTGGCACTAGAAG; GGTAGGCTGAGGTGTAA	NM_009473.3
Malic	Malic enzyme 1	GCCGGCTCTATCCTCCTTTG; TTTGTATGCATCTTGCACAATCTTT	NM_008615.2
Mc4r	Melanocortic 4 receptor	CCCGGACGGAGGATGCTAT; TCGCCACGATCACTAGAATGT	NM_016977.4
Mcad	Medium-chain acyl-CoA dehydrogenase	GCAACTGCCCGCAAGTTT; TACTCCCCGCTTTTGTCATATTC	NM_007382.5
Mgl	Monoglyceride lipase, transcript variant 1	CGGACTTCCAAGTTTTTGTCAGA; GCAGCCACTAGGATGGAGATG	NM_001166251.1
Npy	Neuropeptide Y	CCTTCCATGTGGTGATGGGA; GCAGACTGGTTTCAGGGGAT	NM_023456.3
Npy1r	Neuropeptide Y postsynaptic receptor Y1, transcript variant 1	CACAGGCTGTCTTACACG; GCGAATGTATATCTTGAAGTAG	NM_010934.4
Nr3c1	Nuclear receptor subfamily 3 group C member 1, transcript variant 1	GGAAGCGTGATGGACTTGTAT; GCTTGGAATCTGCCTGAGAA	NM_008173.4
Orexins	Orexins-total	GTCGCCAGAAGACGTGTTC; GGTGGTAGTTACGGTCGGAC	NM_010410.2
Oxt	Oxytocin	TGGCTTACTGGCTCTGACCT; GGCAGGTAGTTCTCCTCCTG	NM_011025.4
Pai	P*ropionibacterium acnes* isomerase	TGACGAGCGGGAATACTTTA; GAGGGTCATCAGCCCATCTA	
Pepck	Phosphoenolpyruvate carboxykinase	CCACAGCTGCTGCAGAACA; GAAGGGTCGCATGGCAAA	NM_011044.3
Pgc1α	Ppar-γ coactivator 1 alpha, transcript variant 1	CCCTGCCATTGTTAAGACC; TGCTGCTGTTCCTGTTTTC	NM_008904.3

Pgd	6-Phosphogluconate dehydrogenase, transcript variant 1	TGAAGGGTCCTAAGGTGGTCC; CCGCCATAATTGAGGGTCCAG	NM_001081274.2
Pgr	Progesterone receptor	CTCCGGGACCGAACAGAGT; ACAACAACCCTTTGGTAGCAG	NM_008829.2
Pi3k	Phosphoinositide 3-kinase, transcript variant 2	GACAGCGAAGCGACGGC; GTCTGATTTTACTGCCACGCTC	NM_001077495.2
Plin1	Perilipin 1a	CTGTGTGCAATGCCTATGAGA; CTGGAGGGTATTGAAGAGCCG	NM_175640.2
Pomc	Proopiomelanocortin, transcript variant 1	CTCCTGCTTCAGACCTCCAT; CAGTCAGGGGCTGTTCATCT	NM_001278581.1
Ppar-γ	Peroxisome proliferator-activated receptor-γ, transcript variant 1	CACAATGCCATCAGGTTTGG; GCTGGTCGATATCACTGGAGATC	NM_001127330.2
Prdm16	PR domain containing 16	GACTTGGACACTACCACGGG; AGATGCACCCCCAAACTCAG	NM_027504.3
Scap	SREBP cleavage-activating protein	ATTTGCTCACCGTGGAGATGTT; GAAGTCATCCAGGCCACTACTAATG	NM_001001144.3
Scd1	Stearoyl-coA desaturase 1	TCCTCCCTACCTCCAACT; CAACAACCAACCCTCGCA	NM_009127.4
Srebp1a	Sterol regulatory element binding protein 1a, transcript variant X1/2	GGCCGAGATGTGCGAACT; TTGTTGATGAGCTGGAGCATGT	NM_011480.4
Srebp1c	Sterol regulatory element binding protein 1c, transcript variant X3	GGAGCCATGGATTGCACATT; GGCCCGGGAAGTCACTGT	NM_001358314.1
Srebp2	Sterol regulatory element binding protein 2	GCGTTCTGGAGACCATGGA; ACAAAGTTGCTCTGAAAACAAATCA	NM_033218.1
Stat3	Signal transducer and activator of transcription 3	CTGTAGAGCCATACACCAAGCAGCAGC; GGTCTTCAGGTACGGGGCAGCAC	NM_213659.3
Ucp1	Uncoupling protein 1	GAGGTGTGGCAGTGTTCATTG; GGCTTGCATTCTGACCTTCA	NM_009463.3
Ucp2	Uncoupling protein 2	GCTTCTGCACCACCGTCAT; GCCCAAGGCAGAGTTCATGT	NM_011671.5

Supplementary Table S3. Fatty acids (mg/g) in wild-type and Pai tissues

	Wild-type	Pai/wt	Pai/Pai
Hearts			
No. of samples	5	4	4
8:0	0.010 ± 0.01	0.011 ± 0.00	0.010 ± 0.01
10:0	ND	ND	ND
12:0	ND	ND	ND
14:0	0.014 ± 0.00[a]	0.015 ± 0.00[ab]	0.019 ± 0.00[b]
14:1	ND	ND	ND
16:0	1.364 ± 0.09[a]	1.441 ± 0.20[ab]	1.676 ± 0.21[b]
16:1n-7	0.041 ± 0.01[a]	0.056 ± 0.01[ab]	0.052 ± 0.00[b]
18:0	1.936 ± 0.25	1.803 ± 0.30	2.160 ± 0.21
trans-18:1	0.016 ± 0.01	0.022 ± 0.01	0.019 ± 0.01
18:1n-9	0.760 ± 0.12[a]	0.810 ± 0.17[ab]	1.010 ± 0.08[b]
18:1n-7	0.287 ± 0.03[a]	0.328 ± 0.06[ab]	0.355 ± 0.04[b]
18:2n-6	1.880 ± 0.23	1.951 ± 0.38	2.120 ± 0.27
20:0	0.008 ± 0.00	0.009 ± 0.00	0.008 ± 0.00
18:3n-6	ND	ND	ND
18:3n-3	0.007 ± 0.00	0.009 ± 0.00	0.009 ± 0.00
t10c12-CLA	ND	ND	ND
20:3n-6	0.112 ± 0.03	0.113 ± 0.03	0.116 ± 0.01
20:4n-6	1.253 ± 0.31	1.256 ± 0.22	1.244 ± 0.16
20:5n-3	0.008 ± 0.00	0.008 ± 0.00	0.009 ± 0.00
24:1	0.053 ± 0.02	0.058 ± 0.00	0.058 ± 0.01
22:5n-3	0.142 ± 0.02	0.151 ± 0.03	0.155 ± 0.02
22:6n-3	2.673 ± 0.37	2.559 ± 0.46	3.087 ± 0.34
Total FAs	11.037 ± 1.09	11.176 ± 1.76	12.750 ± 1.30
SFAs	3.310 ± 0.34	3.252 ± 0.50	3.843 ± 0.41
Unsaturated FAs	7.367 ± 0.69	7.441 ± 1.21	8.368 ± 0.90
MuFAs	1.174 ± 0.15[a]	1.298 ± 0.25[ab]	1.518 ± 0.12[b]
PUFAs	6.104 ± 0.53	6.067 ± 0.97	6.770 ± 0.78
n-6	2.831 ± 0.37	2.724 ± 0.48	3.259 ± 0.36
n-3	3.245 ± 0.56	3.319 ± 0.62	3.480 ± 0.44
n-6/n-3	1.175 ± 0.34	1.230 ± 0.21	1.068 ± 0.06

Supplementary Table S3. Fatty acids (mg/g) in wild-type and Pai tissues

	Wild-type	Pai/wt	Pai/Pai
n7/(n7+16:0)	0.193 ± 0.01	0.210 ± 0.02	0.196 ± 0.01
n7/(n7+18:0)	0.282 ± 0.01[a]	0.309 ± 0.02[ab]	0.319 ± 0.01[b]
(n-6-LA)/n-6	0.417 ± 0.03[ab]	0.413 ± 0.01[a]	0.391 ± 0.01[b]
Livers			
No. of samples	7	5	5
8:0	0.013 ± 0.00[a]	0.007 ± 0.00[b]	0.012 ± 0.00[ab]
10:0	ND	ND	ND
12:0	ND	ND	ND
14:0	0.019 ± 0.01	0.020 ± 0.00	0.016 ± 0.01
14:1	0.009 ± 0.00[ab]	0.007 ± 0.00[a]	0.009 ± 0.00[b]
16:0	1.916 ± 0.30	1.806 ± 0.19	1.654 ± 0.36
16:1n-7	0.121 ± 0.04	0.098 ± 0.02	0.090 ± 0.06
18:0	1.785 ± 0.23[a]	1.736 ± 0.38[ab]	1.465 ± 0.16[b]
trans-18:1	0.023 ± 0.01	0.030 ± 0.02	0.020 ± 0.01
18:1n-9	1.440 ± 0.29	1.382 ± 0.10	1.261 ± 0.31
18:1n-7	0.301 ± 0.11	0.288 ± 0.02	0.221 ± 0.11
18:2n-6	1.350 ± 0.21[a]	1.104 ± 0.21[ab]	1.045 ± 0.19[b]
20:0	0.008 ± 0.00	0.007 ± 0.00	0.008 ± 0.00
18:3n-6	0.019 ± 0.00	0.018 ± 0.00	0.017 ± 0.00
18:3n-3	0.011 ± 0.00	0.009 ± 0.00	0.009 ± 0.00
t10c12-CLA	0.003 ± 0.00	0.004 ± 0.00	ND
20:3n-6	0.238 ± 0.08[a]	0.173 ± 0.04[ab]	0.150 ± 0.04[b]
20:4n-6	1.768 ± 0.27[a]	1.624 ± 0.41[ab]	1.463 ± 0.18[b]
20:5n-3	0.030 ± 0.01[a]	0.019 ± 0.01[b]	0.022 ± 0.01[ab]
24:1	0.027 ± 0.00[a]	0.026 ± 0.01[ab]	0.021 ± 0.00[b]
22:5n-3	0.031 ± 0.01[a]	0.025 ± 0.00[b]	0.024 ± 0.01[ab]
22:6n-3	0.949 ± 0.12	0.923 ± 0.17	0.752 ± 0.16
Total FAs	10.903 ± 0.93[a]	10.668 ± 1.77[ab]	9.116 ± 1.17[b]
SFAs	3.740 ± 0.35[a]	3.576 ± 0.53[ab]	3.154 ± 0.43[b]
Unsaturated FAs	6.460 ± 0.90[a]	5.871 ± 0.69[ab]	5.211 ± 0.81[b]
MuFAs	1.945 ± 0.40	1.865 ± 0.12	1.646 ± 0.45
PUFAs	4.421 ± 0.57[a]	3.934 ± 0.66[ab]	3.502 ± 0.47[b]
n-6	3.375 ± 0.49[a]	2.919 ± 0.53[ab]	2.676 ± 0.32[b]
n-3	1.022 ± 0.13[a]	0.974 ± 0.16[ab]	0.807 ± 0.17[b]

Supplementary Table S3. Fatty acids (mg/g) in wild-type and Pai tissues

	**Wild-type**	**Pai/wt**	**Pai/Pai**
n-6/n-3	3.322 ± 0.45	3.010 ± 0.45	3.373 ± 0.43
n7/(n7+16:0)	0.178 ± 0.04	0.177 ± 0.02	0.152 ± 0.04
n7/(n7+18:0)	0.444 ± 0.05	0.448 ± 0.05	0.458 ± 0.06
(n-6-LA)/n-6	0.600 ± 0.03	0.619 ± 0.05	0.611 ± 0.04
Kidneys			
No. of samples	5	6	4
8:0	0.013 ± 0.01[a]	0.004 ± 0.00[b]	0.016 ± 0.00[a]
10:0	ND	ND	ND
12:0	ND	ND	ND
14:0	0.022 ± 0.00[a]	0.045 ± 0.02[b]	0.021 ± 0.00[a]
14:1	0.007 ± 0.00	0.008 ± 0.00	0.008 ± 0.00
16:0	1.830 ± 0.25[a]	2.654 ± 0.57[b]	1.981 ± 0.13[a]
16:1n-7	0.062 ± 0.02[a]	0.175 ± 0.08[b]	0.062 ± 0.01[a]
18:0	1.703 ± 0.25	2.097 ± 0.35	1.814 ± 0.13
trans-18:1	0.012 ± 0.00	0.008 ± 0.00	0.010 ± 0.00
18:1n-9	0.839 ± 0.18[a]	1.287 ± 0.27[b]	0.904 ± 0.05[a]
18:1n-7	0.306 ± 0.06[a]	0.488 ± 0.07[b]	0.333 ± 0.02[a]
18:2n-6	1.024 ± 0.10[a]	1.394 ± 0.15[b]	1.055 ± 0.08[a]
20:0	0.013 ± 0.00	0.016 ± 0.00	0.014 ± 0.00
18:3n-6	0.009 ± 0.00[a]	0.012 ± 0.00[b]	0.010 ± 0.00[a]
18:3n-3	0.008 ± 0.00[a]	0.015 ± 0.00[b]	0.007 ± 0.00[a]
t10c12-CLA	0.006 ± 0.00[a]	0.011 ± 0.00[b]	0.006 ± 0.00[a]
20:3n-6	0.108 ± 0.02[a]	0.142 ± 0.02[b]	0.099 ± 0.01[a]
20:4n-6	2.226 ± 0.33[a]	2.863 ± 0.50[b]	2.319 ± 0.16[a]
20:5n-3	0.022 ± 0.01[ab]	0.031 ± 0.01[a]	0.019 ± 0.00[b]
24:1	0.051 ± 0.01	0.054 ± 0.01	0.051 ± 0.00
22:5n-3	0.269 ± 0.48[ab]	0.066 ± 0.01[a]	0.049 ± 0.01[b]
22:6n-3	0.937 ± 0.50	1.405 ± 0.42	1.350 ± 0.13
Total FAs	10.15 ± 1.39[a]	13.32 ± 1.69[b]	11.19 ± 1.47[a]
SFAs	3.576 ± 0.50[a]	4.817 ± 0.61[b]	3.845 ± 0.24[a]
Unsaturated FAs	5.999 ± 0.82[a]	8.096 ± 1.06[b]	6.389 ± 0.40[a]
MuFAs	1.283 ± 0.25[a]	2.046 ± 0.37[b]	1.384 ± 0.07[a]
PUFAs	4.633 ± 0.57[a]	5.973 ± 1.07[b]	4.945 ± 0.35[ab]
n-6	3.367 ± 0.43[a]	4.411 ± 0.65[b]	3.483 ± 0.24[a]

Supplementary Table S3. Fatty acids (mg/g) in wild-type and Pai tissues

	Wild-type	Pai/wt	Pai/Pai
n-3	1.231 ± 0.17	1.516 ± 0.43	1.426 ± 0.13
n-6/n-3	2.743 ± 0.21[a]	2.995 ± 0.37[a]	2.450 ± 0.14[b]
n7/(n7+16:0)	0.167 ± 0.01[a]	0.199 ± 0.02[b]	0.166 ± 0.01[a]
n7/(n7+18:0)	0.329 ± 0.04	0.381 ± 0.07	0.333 ± 0.01
(n-6-LA)/n-6	0.695 ± 0.02	0.682 ± 0.02	0.697 ± 0.01
Skeletal muscle			
No. of samples	4	4	4
8:0	0.035 ± 0.01	0.031 ± 0.02	0.025 ± 0.02
10:0	ND	ND	ND
12:0	0.028 ± 0.01	0.014 ± 0.01	0.009 ± 0.00
14:0	0.039 ± 0.01	0.035 ± 0.02	0.169 ± 0.29
14:1	ND	ND	ND
16:0	1.200 ± 0.21	0.887 ± 0.48	0.942 ± 0.48
16:1n-7	0.117 ± 0.03[a]	0.132 ± 0.10[ab]	0.075 ± 0.02[b]
18:0	0.723 ± 0.10	0.450 ± 0.24	0.582 ± 0.33
trans-18:1	ND	ND	ND
18:1n-9	0.398 ± 0.07	0.412 ± 0.31	0.326 ± 0.21
18:1n-7	0.167 ± 0.03	0.119 ± 0.07	0.130 ± 0.07
18:2n-6	1.229 ± 0.25[a]	1.048 ± 0.32[ab]	0.687 ± 0.20[b]
20:0	ND	ND	ND
18:3n-6	ND	ND	ND
18:3n-3	ND	ND	ND
t10c12-CLA	ND	ND	ND
20:3n-6	ND	ND	ND
20:4n-6	0.685 ± 0.08	0.482 ± 0.26	0.581 ± 0.45
20:5n-3	ND	ND	ND
24:1	ND	ND	ND
22:5n-3	0.078 ± 0.03	0.048 ± 0.02	0.054 ± 0.02
22:6n-3	0.944 ± 0.35	0.593 ± 0.25	0.705 ± 0.12
Total FAs	6.423 ± 1.17	5.043 ± 2.37	5.833 ± 2.25
SFAs	2.071 ± 0.34	1.458 ± 0.78	1.775 ± 0.75
Unsaturated FAs	3.624 ± 0.70	2.842 ± 1.26	2.589 ± 1.06
MuFAs	0.683 ± 0.13	0.663 ± 0.47	0.530 ± 0.29
PUFAs	2.941 ± 0.64	2.170 ± 0.80	2.048 ± 0.77

Supplementary Table S3. Fatty acids (mg/g) in wild-type and Pai tissues

	Wild-type	Pai/wt	Pai/Pai
n-6	1.914 ± 0.26	1.530 ± 0.56	1.283 ± 0.64
n-3	1.027 ± 0.38	0.641 ± 0.26	0.765 ± 0.14
n-6/n-3	1.973 ± 0.41	2.472 ± 0.57	1.618 ± 0.49
n7/(n7+16:0)	0.192 ± 0.02[ab]	0.213 ± 0.02[a]	0.182 ± 0.02[b]
n7/(n7+18:0)	0.354 ± 0.04	0.454 ± 0.08	0.351 ± 0.03
(n-6-LA)/n-6	0.362 ± 0.05	0.305 ± 0.05	0.431 ± 0.09
BAT			
No. of samples	4	4	4
8:0	0.012 ± 0.00	0.011 ± 0.00	ND
10:0	ND	ND	ND
12:0	0.012 ± 0.00[a]	0.011 ± 0.00[a]	0.016 ± 0.00[b]
14:0	0.168 ± 0.02	0.170 ± 0.02	0.216 ± 0.07
14:1	0.012 ± 0.00[ab]	0.011 ± 0.00[a]	0.016 ± 0.00[b]
16:0	2.559 ± 0.21	2.713 ± 0.44	3.354 ± 1.36
16:1n-7	0.451 ± 0.09	0.452 ± 0.17	0.717 ± 0.19
18:0	1.372 ± 0.20	1.367 ± 0.39	1.371 ± 0.45
trans-18:1	0.096 ± 0.05	0.093 ± 0.06	0.080 ± 0.05
18:1n-9	3.177 ± 0.90	2.763 ± 0.81	3.420 ± 1.33
18:1n-7	0.610 ± 0.17	0.612 ± 0.15	0.824 ± 0.29
18:2n-6	1.878 ± 0.33	1.606 ± 0.37	1.683 ± 0.40
20:0	ND	ND	ND
18:3n-6	0.027 ± 0.01	0.021 ± 0.00	0.024 ± 0.01
18:3n-3	0.033 ± 0.01	0.028 ± 0.01	0.035 ± 0.01
t10c12-CLA	0.022 ± 0.02	0.029 ± 0.01	0.030 ± 0.00
20:3n-6	0.062 ± 0.00	0.066 ± 0.01	0.064 ± 0.01
20:4n-6	1.121 ± 0.28	1.135 ± 0.30	1.058 ± 0.21
20:5n-3	0.015 ± 0.00	0.017 ± 0.00	0.020 ± 0.00
24:1	0.023 ± 0.00	0.025 ± 0.01	0.028 ± 0.01
22:5n-3	0.022 ± 0.00	0.023 ± 0.00	0.022 ± 0.01
22:6n-3	0.342 ± 0.03	0.354 ± 0.07	0.317 ± 0.09
Total FAs	15.205 ± 3.09	14.043 ± 3.81	15.710 ± 5.56
SFAs	4.122 ± 0.36	4.270 ± 0.81	4.957 ± 1.88
Unsaturated FAs	8.050 ± 1.78	7.353 ± 1.67	8.455 ± 2.57
MuFAs	4.421 ± 1.18	3.991 ± 1.17	5.127 ± 1.83

Supplementary Table S3. Fatty acids (mg/g) in wild-type and Pai tissues

	Wild-type	Pai/wt	Pai/Pai
PUFAs	3.550 ± 0.60	3.285 ± 0.76	3.258 ± 0.73
n-6	3.089 ± 0.61	2.828 ± 0.68	2.829 ± 0.62
n-3	0.412 ± 0.03	0.421 ± 0.08	0.395 ± 0.10
n-6/n-3	7.586 ± 2.01	6.687 ± 0.58	7.256 ± 0.73
n7/(n7+16:0)	0.291 ± 0.05	0.279 ± 0.06	0.324 ± 0.06
n7/(n7+18:0)	0.693 ± 0.03	0.663 ± 0.10	0.711 ± 0.02
(n-6-LA)/n-6	0.390 ± 0.02	0.432 ± 0.01	0.407 ± 0.02

Note: Tissues of wild-type, Pai/wt, and Pai/Pai mice at the age of 11 weeks are for analysis, and 19:0 is an internal standard. Each value represents the mean ± SD. ND, Not detected; LA, linoleic acid; FA, fatty acids. Total FAs are total amounts from 8:0 to 22:6n-3 compositions. SFAs (saturated) and MUFAs (monounsaturated) are calculated as 8:0 + 10:0 + 12:0 + 14:0 + 16:0 + 18:0 + 20:0 + 22:0 + 24:0, and 14:1 + 16:1n-7 + trans-18:1 + 18:1n-9 + 18:1n-7 + 20:1 + 22:1 + 24:1, respectively. PUFAs is calculated as n-6 + n-3 as well as n-6 and n-3 are calculated as 18:2n-6 + 18:3n-6 + 20:3n-6 + 20:4n-6, and 18:3n-3 + 20:5n-3 + 22:5n-3 + 22:6n-3, respectively. n-7/(n-7 + 16:0) and n-7/(n-7 + 18:0) are calculated as (16:1n-7 + 18:1n-7)/(16:1n-7 + 18:1n-7 + 16:0), and 18:1n-9/(18:1n-9 + 18:0), respectively. Different letters in superscript indicate $p < 0.05$.

Supplementary Table S4. Dissection analysis of wild-type and Pai mice

	wt	Pai/wt	Pai/Pai
Number of mice	21	17	13
Bodyweight (g)	21.6 ± 1.8	22.0 ± 1.6	22.1 ± 1.6
Heart weight (%)	0.49 ± 0.03[a]	0.51 ± 0.03[ab]	0.55 ± 0.08[b]
Liver weight (%)	4.78 ± 0.61[a]	4.96 ± 0.55[ab]	5.30 ± 0.58[b]
Spleen weight (%)	0.43 ± 0.08[a]	0.43 ± 0.07[a]	0.55 ± 0.09[b]
Kidney weight (%)	1.22 ± 0.12	1.27 ± 0.10	1.18 ± 0.37
Lung weight (%)	0.65 ± 0.10	0.63 ± 0.09	0.70 ± 0.13
Pancreas weight (%)	0.56 ± 0.08	0.52 ± 0.07	0.59 ± 0.10
Brain weight (%)	1.44 ± 0.15	1.41 ± 0.16	1.37 ± 0.13
Hypothalamus weight (%)	0.04 ± 0.01	0.04 ± 0.02	0.05 ± 0.02
Ovary weight (%)	0.04 ± 0.01[a]	0.05 ± 0.01[b]	0.05 ± 0.01[b]
WAT weight (%)	0.35 ± 0.14	0.34 ± 0.16	0.32 ± 0.14
BAT weight (%)	0.41 ± 0.10	0.43 ± 0.09	0.41 ± 0.09
Small intestine length (cm)	35.5 ± 1.9	35.6 ± 2.2	37.0 ± 2.7

Note: Mice at 11 weeks of age were killed in the non-fasted state, and their issue/organ weight as a percentage of slaughter body weight was calculated. BAT, interscapular brown adipose tissue; WAT, peri-gonadal white adipose tissue. Each value represents the mean ± SD. Different letters in superscript indicate $p < 0.05$.

Supplementary Figures.

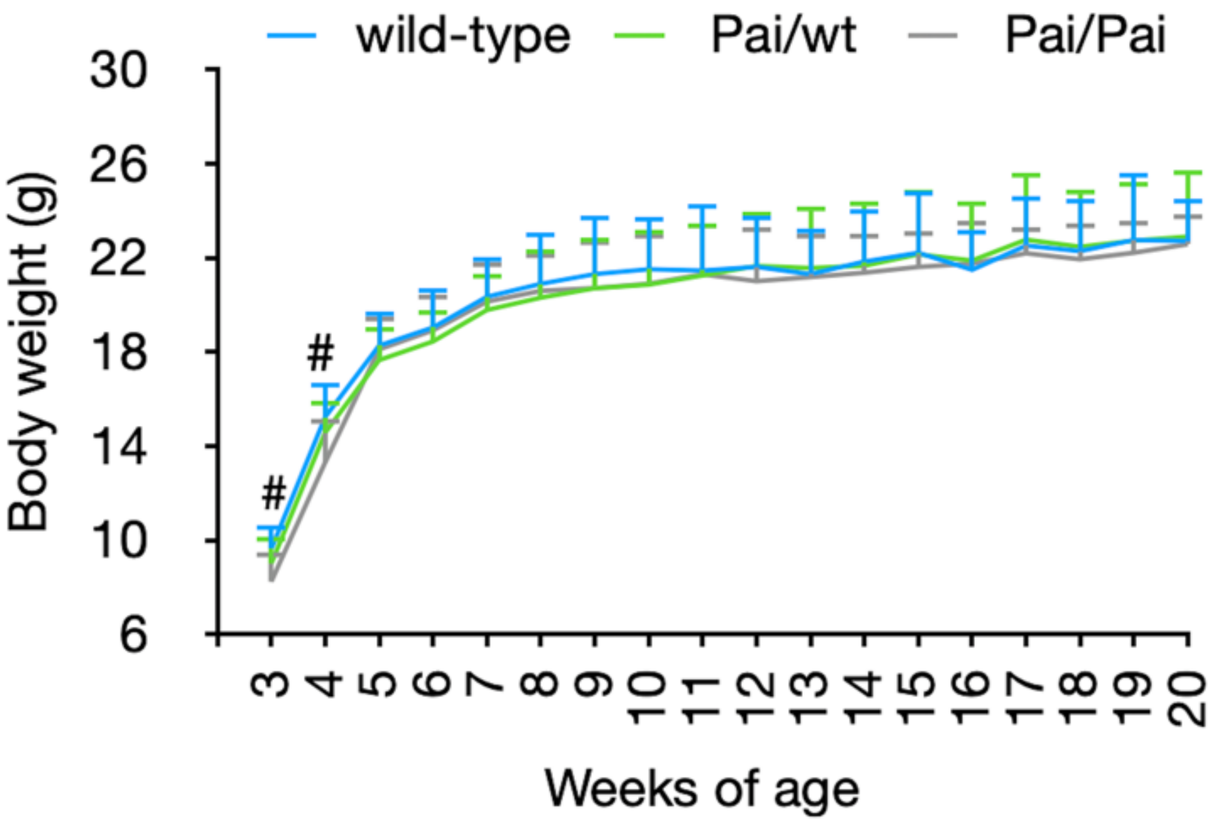

Supplementary Figure S1. Comparison of growth of wild-type and Pai mice. Each group contained 11-29 mice. Bars represent the mean ± SD. # indicates $p < 0.05$ among three genotypes.

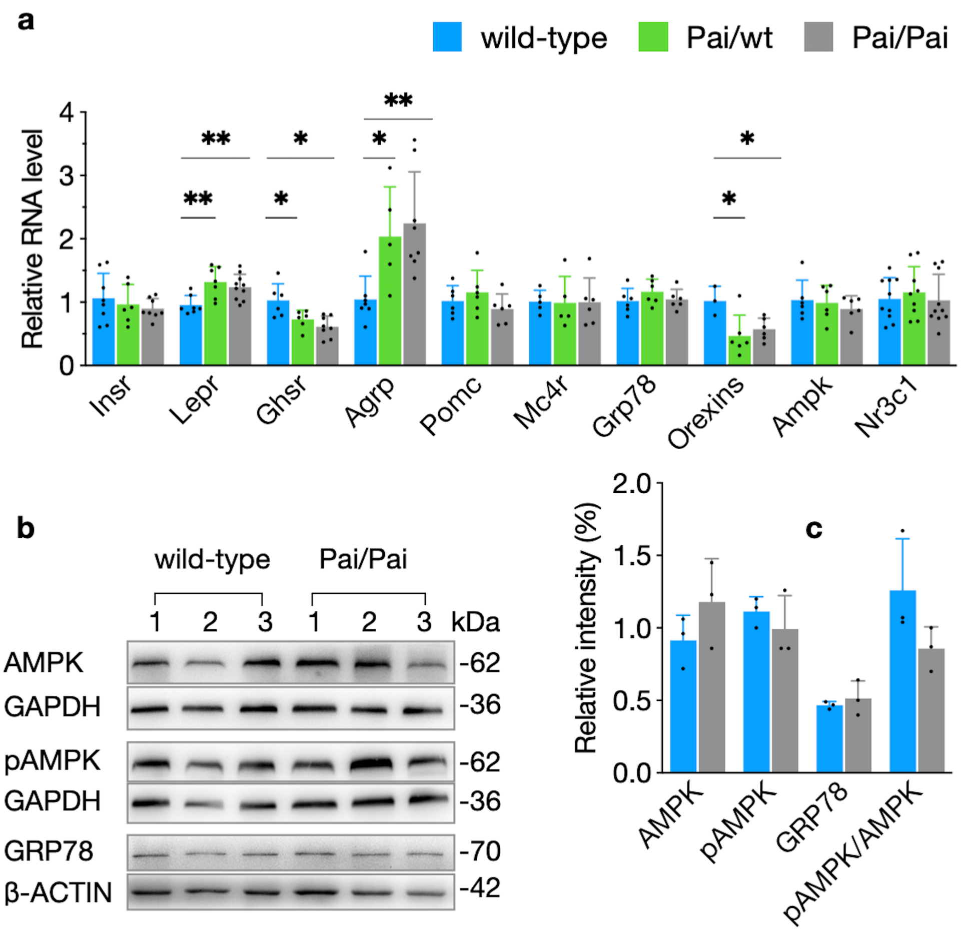

Supplementary Figure S2. Analyses of the relative mRNAs (a), protein levels and their relative intensities in the hypothalamus (b-c). Agrp, Agouti-related peptide; Ampk, AMP-activated protein kinase; Ghsr, Ghrelin receptor; Grp78, glucose-regulated protein 78; Insr, Insulin receptor; Lepr, Leptin receptor; Mc4r, Melanocortic 4 receptor; Nr3c1, Nuclear receptor subfamily 3 group C member 1; (p)AMPK, (phosphorylated) AMP-activated protein kinase; Pomc, Proopiomelanocortin. The bars represent the mean ± SD. * indicates $p < 0.05$ and ** indicates $p < 0.01$, respectively.

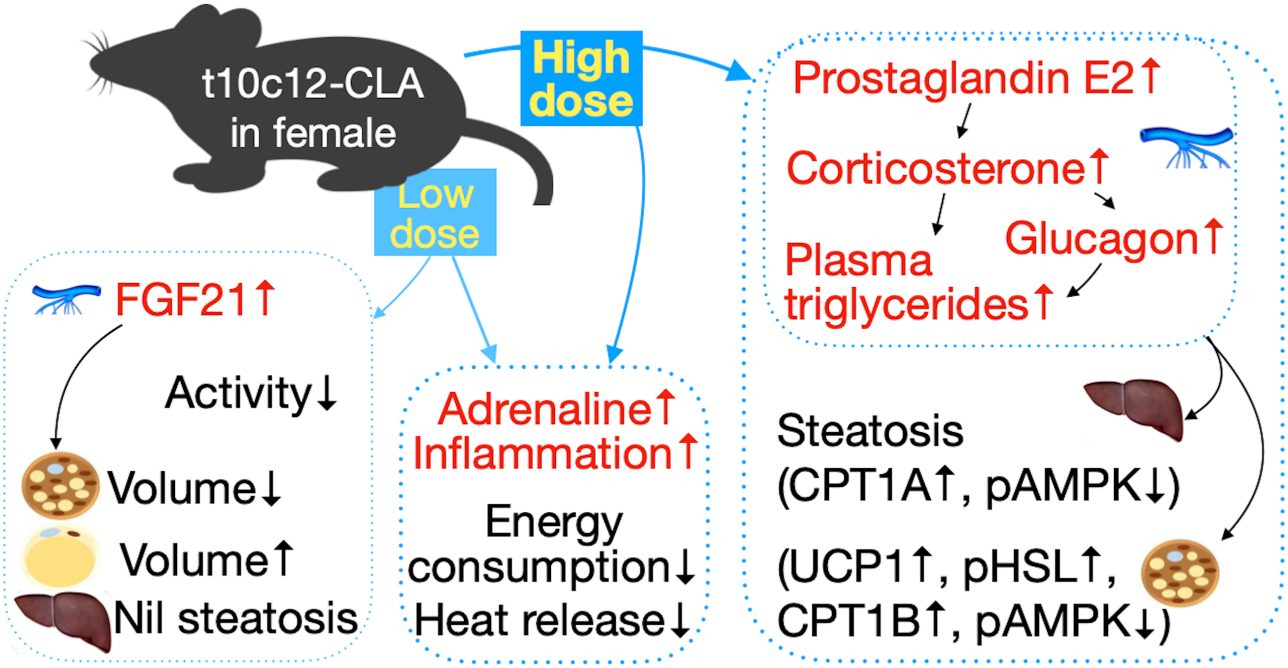

Supplementary Figure S3. Diagram showing the dose-(in)dependent effect of chronic t10c12-CLA on lean female mice. Besides the common (dose-independent) effects, such as increased adrenaline, inflammation reaction, less heat production, and low energy consumption (Middle panel), the low doses specifically increase circulating levels of FGF21, which plays an active role in maintaining energy homeostasis and suppress physical activity in Pai/wt mice (Left panel). Conversely, the high doses specifically result in excesses of prostaglandin E2, corticosterone, and glucagon; subsequently, the combination of these factors causes metabolic syndrome, such as fatty liver and hypertriglyceridemia through affecting the activities of critical enzymes involved in lipid metabolism in livers (CPT1A, pAMPK, etc.) and adipose tissues (UCP1, pHSL, CPT1B, pAMPK etc.) of Pai/Pai mice (Right panel). Words in black and red describe physiological features and endocrinal factors, respectively.
